# Virtual Reality and Eye-Tracking Assessment, and Treatment of Unilateral Spatial Neglect: Systematic Review and Future Prospects

**DOI:** 10.3389/fpsyg.2022.787382

**Published:** 2022-03-22

**Authors:** Alexander Pilgaard Kaiser, Kristian Westergaard Villadsen, Afshin Samani, Hendrik Knoche, Lars Evald

**Affiliations:** ^1^Hammel Neurorehabilitation Centre and University Research Clinic, Hammel, Denmark; ^2^Department of Psychology and Behavioral Sciences, Aarhus University, Aarhus, Denmark; ^3^Aalborg University Hospital, Aalborg University, Aalborg, Denmark; ^4^Department of Health Science and Technology, Aalborg University, Aalborg, Denmark; ^5^Department of Architecture, Design and Media Technology, Aalborg University, Aalborg, Denmark; ^6^Department of Clinical Medicine, Faculty of Health, Aarhus University, Aarhus, Denmark

**Keywords:** eye-tracking (ET), unilateral spatial neglect (USN), assessment, treatment, immersive virtual reality (VR), visuospatial disorders, attention

## Abstract

Unilateral spatial neglect (USN) is a disorder characterized by the failure to report, respond to, or orient toward the contralateral side of space to a brain lesion. Current assessment methods often fail to discover milder forms, cannot differentiate between unilateral spatial neglect subtypes and lack ecological validity. There is also a need for treatment methods that target subtypes. Immersive virtual reality (VR) systems in combination with eye-tracking (ET) have the potential to overcome these shortcomings, by providing more naturalistic environments and tasks, with sensitive and detailed measures. This systematic review examines the state of the art of research on these technologies as applied in the assessment and treatment of USN. As we found no studies that combined immersive VR and ET, we reviewed these approaches individually. The review of VR included seven articles, the ET review twelve. The reviews revealed promising results. (1) All included studies found significant group-level differences for several USN measures. In addition, several studies found asymmetric behavior in VR and ET tasks for patients who did not show signs of USN in conventional tests. Particularly promising features were multitasking in complex VR environments and detailed eye-movement analysis. (2) No VR and only a few ET studies attempted to differentiate USN subtypes, although the technologies appeared appropriate. One ET study grouped USN participants using individual heatmaps, and another differentiated between subtypes on drawing tasks. Regarding (3) ecological validity, although no studies tested the prognostic validity of their assessment methods, VR and ET studies utilized naturalistic tasks and stimuli reflecting everyday situations. Technological characteristics, such as the field of view and refresh rate of the head-mounted displays, could be improved, though, to improve ecological validity. We found (4) no studies that utilized VR or ET technologies for USN treatment up until the search date of the 26th of February 2020. In conclusion, VR-ET-based systems show great potential for USN assessment. VR-ET holds great promise for treatment, for example, by monitoring behavior and adapting and tailoring to the individual person’s needs and abilities. Future research should consider developing methods for individual subtypes and differential diagnostics to inform individual treatment programs.

## Introduction

More sensitive and accurate assessment methods that reflect the full spectrum of unilateral spatial neglect (USN) including its milder forms and further differentiation between subtypes and differential diagnosis are needed. As are more effective treatment methods that can be tailored to the individual patient’s USN subtypes, combined with different treatment strategies, while increasing the specificity and intensity of the training, e.g., in self- or telerehabilitation.

Novel immersive virtual reality (VR) applications incorporating eye-tracking (ET) may in the near future provide such an opportunity if existing knowledge of conventional assessment and treatment of USN are integrated into their design. Capturing detailed temporal and spatial data on a millisecond-level across the entire visual field while allowing for full-body movements when performing functional tasks may increase the sensitivity and specificity of USN assessment. Immersive VR may also improve ecological validity by providing naturalistic environments and tasks while maintaining the rigorous control of standardized testing and more complex computerized measurements (Parsons, 2016). Meta-analytical research on neuropsychological assessment has documented the sensitivity of detecting impairments across cognitive domains in VR (Negu et al., 2016).

USN is a neurological disorder defined as the failure to report, respond to, or orient toward stimuli located to the contralateral side of a brain lesion, when this failure cannot be explained by sensory or motor deficits (Heilman et al., 2012). Spatial neglect is a common impairment following stroke affecting at least 30% of stroke survivors (Hammerbeck et al., 2019) though the prevalence differs considerably depending on the used assessment methods (Bowen et al., 1999; Chen et al., 2015).

Despite USN being a common impairment following acquired brain injury, milder forms are often underdiagnosed and consequently undertreated (Bowen et al., 1999; Edwards et al., 2006; Chen et al., 2013). Furthermore, USN constitutes a heterogeneous disorder with many different dissociable subtypes that may be distinguished by:

1. The “modality of symptoms,” e.g., the sensory-attentional spatial bias of visual, auditory and tactile input, the motor-intentional spatial bias of movement in or toward the neglected hemispace, and representational neglect in mental imagery.
2. The “range of space” of the attentional spatial bias, e.g., confined to the space of the body (personal neglect), within arm’s reach (peripersonal neglect), or beyond arm’s reach (extrapersonal neglect).
3. The “frame of reference” of the attentional bias in relation to (a) different body midlines such as the trunk, head, or eyes (egocentric neglect) or (b) the midline of objects (allocentric neglect) (Buxbaum et al., 2004; Kerkhoff and Schenk, 2012; Rode et al., 2017).

These subtypes respond differently to treatment approaches (Luaute et al., 2006; Kerkhoff and Schenk, 2012; Azouvi et al., 2017). Thus, accurate identification of USN symptoms and differential diagnostics of USN subtypes are paramount to informing treatment choices. However, conventional assessment methods often prove inadequate and unsatisfactory in this respect. Consequently, there is a need for improving the methods for USN subtype diagnostics and for developing more effective treatment methods that can target specific USN subtypes, which might be achieved by the use of VR and ET.

### Conventional Assessment of Unilateral Spatial Neglect

Conventional assessments most often involve paper-and-pencil tests, but often yield unsatisfactory specificity (true negatives). Due to profound ceiling effects they often fail to detect milder USN in patients who show signs of neglect in activities of daily living (ADL) (Ting et al., 2011). Even though conventional tests often have satisfactory sensitivity (true positives) they cannot reliably distinguish between different USN subtypes. They most often solely encompass the peri-personal space involving visuomotor skills, thus not assessing USN affecting other parts of space, other modalities, or distinguishing between motor and sensory USN (Plummer et al., 2003).

Conventional tests often have limited ecological validity (Azouvi et al., 2006), which is problematic as it limits the ability to predict deficits that are present in everyday situations (Coolican, 2009) and thereby the transferability between the deficits that are assessable in test and everyday situations. The tests lack verisimilitude – their similarity to relevant tasks in real life – since they primarily use static stimuli in artificially controlled environments. Likewise, the veridicality (Chaytor and Schmitter-Edgecombe, 2003) – their ability to predict performance in real life – is limited since patients can perform normally in tests due to ceiling effects or compensatory strategies and still show USN in ADL (Azouvi et al., 2006).

### Ecological Assessment of Unilateral Spatial Neglect

Ecological assessment methods have been designed, but none of them have succeeded in capturing the complexity of USN (Azouvi, 2017). The most promising and widely used ecological assessment option is the Catherine Bergego Scale (CBS; Bergego et al., 1995) and the systematic observation of the CBS elaborated in the Kessler Foundation Neglect Assessment Process (KF-NAP; Chen et al., 2015). The CBS and KF-NAP are observational tools that assess the patient in 10 different ADL categories, including e.g., gaze orientation, limb awareness, and navigating (Azouvi et al., 2006). CBS is considered the most sensitive USN assessment available and often detects USN in patients not assessable by pen-and-paper (Bartolomeo, 2014). The CBS allows for distinguishing between behaviors in different ranges of space but fails to discriminate between motor and sensory USN (Ting et al., 2011). Additionally, it involves relatively easy tasks in rehabilitation settings (Grattan and Woodbury, 2017) and constitutes an inherently subjective measure of the rater, i.e., the health care professional. Some of these limitations may be overcome by VR-ET systems.

### Virtual Reality Assessment of Unilateral Spatial Neglect

Virtual reality (VR) is a user-computer interface involving stimulation and interactions in real-time through multiple sensory channels of an embedded subject. It is based on a synthetic environment where the subject feels present (Burdea and Coiffet, 2003). Complex virtual environments e.g., resembling cluttered kitchens (Cipresso et al., 2014) or grocery stores (Ogourtsova et al., 2018c) allow for assessing natural attentional behavior more accurately than pen-and-paper tests. Moreover, real-world settings pose a danger to patients, such as street-crossings (Kim et al., 2007; Navarro et al., 2013), wayfinding, or driving could be safely simulated in VR.

Immersive VR refers to both CAVE Automatic Virtual Environments (CAVE) and head-mounted displays (HMD). As opposed to non-immersive VR (i.e., technologies with a limited field of view and 2-dimensional screen features), they provide unique advantages, including both higher verisimilitude and sense of presence facilitating naturalistic behavior and limiting confounding factors by shutting out physical reality (Slater and Wilbur, 1997). The main technological features that facilitate presence include the afforded field of view, degrees of freedom, and visual refresh rate (Cummings and Bailenson, 2016).

### Eye-Tracking Assessment of Unilateral Spatial Neglect

In research on eye movements in USN patients, patients tend to produce fewer of a given eye movement type in their contralesional visual field (Fanthome and Lincoln, 1995; Kortman and Nicholls, 2016) such as saccades (Gainotti et al., 2009), gaze and eye position relative to the head (Karnath, 1998) or fixations (Müri et al., 2009) during both visual search and free-viewing (Fruhmann Berger et al., 2008). One case study suggested that eye movement distribution is altered by the amount of stimuli and amount of distractors (Husain et al., 2001). Also, eye-tracking research has facilitated the discussion about the nature of allocentric neglect and the role of eye movements and placement of objects (Karnath et al., 2011; Gainotti and Ciaraffa, 2013). Studies suggested that biased eye movements also underlie pathological performances on conventional USN assessment tools such as line bisection (Ishiai et al., 2006; Chiba et al., 2008; Balconi et al., 2013). From a differential diagnostic point-of-view stroke patients have fewer saccades than healthy (Alves et al., 2016) but presumably, USN patients have smaller (Gainotti et al., 2009) and slower saccades (Butler et al., 2009) as is their gaze and eye placement relative to their head further right compared to healthy and stroke patients (Karnath et al., 1998). Hence, these technologies seem to have untapped potentials in the assessment of USN providing knowledge about visual attentional biases that presumably underly impaired ADL activity (Mort and Kennard, 2003; Müri et al., 2009; Cameirão et al., 2016; Delazer et al., 2018) especially due to an automatic collection of data with high granularity albeit practical issues of calibration and head restraining need addressing (Trepagnier, 2002).

### Aim of the Present Study

Previous systematic reviews have focused on the implication of eye movement training in rehabilitation of USN (Bowen et al., 2013; Hill et al., 2015; Hanna et al., 2017b) emphasizing compensatory strategies (e.g., the light tower), prismatic adaption training, or smooth pursuit training. In assessment, a review found that clinicians predominantly rely on either classical paper-pen tests, neurological examinations, or computer-based versions of classical tests (Hanna et al., 2017a). Hence, it is unclear to what extent ET is currently able to enhance the current assessment and treatment of USN in which eye movements already are assessed to some extent. Further, it is relevant to consider the veridicality in findings from ET studies, which has not been an aim in the previously mentioned reviews.

Unlike previous reviews of VR assessment of USN (e.g., Pedroli et al., 2015; Ogourtsova et al., 2017) the present review focuses solely on immersive VR due to its potential to deliver high verisimilitude and veridicality. To the best of our knowledge, no prior study has combined immersive VR and ET in the assessment or treatment of USN. Therefore, we review these two technologies separately to compile the current evidence for future development potential and research.

The aim is to critically review the state of research on immersive VR and ET applied to the assessment and treatment of USN, seeking answers to four substantial clinical questions: Can immersive VR and ET:

1. reliably detect USN on a group (case/control) level?
2. distinguish USN subtypes on an individual patient level?
3. improve the ecological validity of USN assessment?
4. effectively be applied to USN treatment?

Finally, we discuss the future perspectives on the assessment and treatment of USN by the use of immersive VR and ET.

## Methods

To ensure quality and transparency, our analysis followed the Preferred Reporting Items for Systematic Reviews and Meta-Analysis (PRISMA) guidelines where applicable (Moher et al., 2009).

### Search Strategy

Studies included in this review were identified by searching PsycINFO, PubMed, and Embase using relevant variants, acronyms, and synonyms of the search words “unilateral spatial neglect” and “virtual reality” or “eye-tracking” in the abstract or title and appropriate index terms. Supplementary Material 1 features an overview of this search. The search was carried out on the 26th of February 2020, hence studies added to the databases until this date were included.

For the VR search, additional articles were identified by checking references in reviews by Tsirlin et al. (2009), Pedroli et al. (2015), and Ogourtsova et al. (2017).

### Inclusion and Exclusion Criteria

Table 1 features a patients, intervention, control, and outcome (PICO) overview of the objectives. In the inclusion criteria of both reviews, the study must possess the following:

**TABLE 1:** Patients, intervention, control, and outcome (PICO) overview.

Patients	Intervention	Control	Outcome
Stroke patients with Unilateral Spatial Neglect (USN)	Immersive Virtual Reality and/or Eye tracking	*Assessment studies:* Healthy controls or patients without USN	A measure of any type of eye movements and/or movements/behavior in Virtual reality
		*Treatment studies:* patients with USN

(1) be an empirical peer-reviewed article, published in English, with results not previously published,
(2) include patients with USN as the study population,
(3) assess patients for USN through traditional pen-and-paper tests and/or through functional independence or performance in ADL,
(4) provide the USN assessment results, demographic, and disease-specific information.

For the VR review, the following criteria required the study to

(5a) use immersive VR (CAVE or HMD) as part of the study design,
(6a) use participants with USN and at least one control group or at least two groups of USN patients receiving different treatments,
(7a) provide information about measures used to assess USN in VR, and
(8a) be published after the year 2000, due to technological advances.

Studies including acute as well as chronic USN patients were included, as this should not influence the suitability of the VR system.

For the ET review, the studies had to

(5b) use video-based ET technology,
(6b) present participants with at least one type of naturalistic stimuli. We considered a stimulus to be naturalistic if the scene was like the real environment in daily life and/or the VR task required interactions with objects similar to daily life. Hence, arbitrary shapes like dots or squares were not considered naturalistic since they are not common objects featured in everyday life. This criterion followed the emphasis on immersion and presence in VR and its influence on the possible transfer of training,
(7b) rely on test subjects consisting of neglected patients and at least one control group or at least two groups of neglect patients receiving different treatments,
(8b) provide a clear description of what eye movements are measured and how they are analyzed in relation to USN. Studies with solely reaction time of eye movements were excluded since they do not analyze eye movements *per se*,
(9b) present group comparisons and/or correlational analysis on conventional measures of neglect in the analysis of eye movements. As opposed to the VR literature search, no cut-off publishing year was applied.

### Quality Assessment and Calculation of Effect Sizes

Quality assessment was performed using the appraisal tool for cross-sectional studies (AXIS) (Downes et al., 2016). It consists of 20 items, covering reporting (1, 4, 10, 11, 12, 16, 18), study design (2, 3, 5, 8, 17, 19, 20), and risk of bias (6, 7, 9, 13, 14, 15). Question 14 regarding potential non-responders was excluded because it was not applicable to any included study. Studies were scored on each criterion with 1 if it was fulfilled - for a maximum AXIS score of 19 points.

Hedges *g* was calculated, using an online effect size calculator (Lenhard and Lenhard, 2016) constituting small (*g* ≥ 0.2), medium (*g* ≥ 0.5), or large effects (*g* ≥ 0.8). Hedges *g* shares similarities to Cohen’s *d* but applies a pooled standard deviation (see Eq. 1) (Nakagawa and Cuthill, 2007). Correlations between results and conventional assessment are reported in Pearson’s *r*, whenever this was provided by the study. If only other measures of error rate such as variance or standard error of mean were reported the standard deviation was calculated manually.


(1) $$Hedge^{\prime }sg=\frac{m_{2}-m_{1}}{\sqrt{\frac{\left(n2-1\right)SD2^{2}+\left(n1-1\right)SD1^{2}}{n1+n2-2}}}$$

## Virtual Reality Review

### Virtual Reality Results

#### Study Selection

**FIGURE 1:**
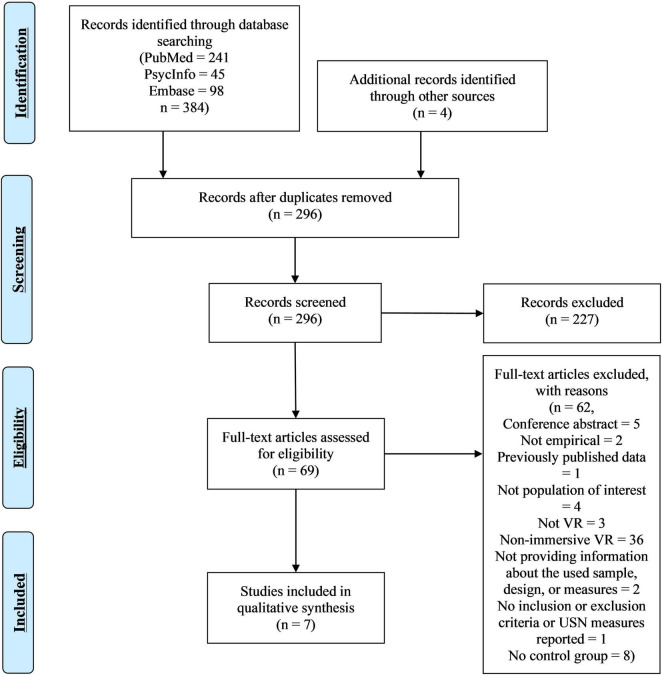
Virtual reality (VR) search flow chart.

In total, 384 records were identified. Removing duplicates and screening previous reviews for references left 296 records. Titles and abstracts were screened using the inclusion criteria from section 2.2, leaving 69 records for full-text screening for eligibility. Seven studies were included in the review (see Figure 1 for an overview).

#### Quality Assessment

Appraisal tool for cross-sectional studies (AXIS)-scores ranged from 12 to 17 (M = 15.4, SD = 1.62) (see Table 2). Only one study justified their sample size (Ogourtsova et al., 2018b), suggesting 13 participants per group. The risk of bias was revealed, as only two studies (Kim et al., 2010; Aravind and Lamontagne, 2017) included a selection process that was likely to sample participants representative of the target population. Three studies included patients with a history of USN, i.e., no current symptoms, in the patient group (Ogourtsova et al., 2018a,b,c), and one study included patients with scores above the USN cutoffs in the traditional tests and did not screen for cognitive deficits (Peskine et al., 2011). One study did not report any inclusion or exclusion criteria (Kim et al., 2004). In addition, all studies were the first published use of the assessment instruments and were not previously piloted. Scores for each AXIS item are presented in Supplementary Material 2.

**TABLE 2:** Virtual reality (VR) general characteristics.

Authors	USN^1^ participants *N* (male/female)	USN participants Mean age (*SD*^2^)	Type of controls; *N* (male/female)	Control group Mean age (*SD*)	Conventional USN assessment; Mean USN patient score (*SD*)	Cognitive screening (cut-off score); Mean USN patient score (*SD*)	Equipment	VR^2^ characteristics	AXIS^15^-score/19
Aravind and Lamontagne, 2017	13 (N/A)	59.8 (7.7)	Non-USN^3^; 13 (N/A)	60.8 (6.5)	BCT^4^; 6.9 (2.1) omissions ACT^5^; 5.2 (2.5) omissions LBT^6^; 103.8 (51.2)*	MoCA^7^ (N/A); 26.1 (1.5)	nVisor SX60 HMD^1^ (1024 × 1280 pixels; 60° diagonal field of view); Vicon-512 Motion capture system	Head and body tracking	16
Kim et al., 2004	12 (8/4)	54.9 (17.4)	HC^8^ with high PC experience; 20 (15/5) HC with low PC experience; 20 (5/15)	1: 29.5 (2.5) 2: 59.9 (6.1)	LBT; 37.5 (27.77)* LCT^9^; 34.6 (31.4)*	MMSE^10^ (>21), CPM^11^, WMS^12^; N/A	Eye-track FMD-250W HMD; Intertrax2 Position Sensor with 3 degrees of freedom	Head-tracking	12
Kim et al., 2010	16 (10/6)	52.9 (16.8)	Non-USN; 16 (11/5)	60.1 (12.1)	LBT; 31.7 (19.2)% deviation LCT; 21.9 (18.6)% missing	N/A	Unspecified HMD; Head-tracking system with 3 degrees of freedom	Head-tracking; mouse button responses	16
Ogourtsova et al., 2018a	15 (12/3)	60.2 (8.8)	Non-USN; 15 (13/2) HC; 15 (7/8)	1: 58.5 (13.2) 2: 61 (11.3)	LBT, near; 0.9 (0.7) cm deviation LBT, far; 6.7 (6.2) cm deviation SCT^13^, near; 0.9 (0.11) canceled/total SCT, far; 0.9 (0.09) canceled/total ACT; 0.9 (0.08) canceled/total	MoCA (≤22); N/A	NVisor HMD (1024 × 1280 pixels, 60 Hz refresh rate, 60° diagonal field of view); Attack3 Logitech Joystick	No head-tracking; Joystick responses	16
Ogourtsova et al., 2018b	15 (12/3)	60.2 (8.8)	Non-USN; 15 (13/2) HC; 15 (7/8)	1: 58.5 (13.2) 2: 61 (11.3)	LBT, near; 0.9 (0.7) cm deviation LBT, far; 6.7 (6.2) cm deviation SCT, near; 0.9 (0.11) canceled/total SCT, far; 0.9 (0.09) canceled/total ACT; 0.9 (0.08) canceled/total	MoCA (≤22); N/A	NVisor HMD (1024 × 1280 pixels, 60 Hz refresh rate; 60° diagonal field of view); Vicon-512 Motion capture system	Head and body tracking	17
Ogourtsova et al., 2018c	12 (9/3)	60.7 (9.1)	Non-USN; 15 (13/2) HC; 9 (4/5)	1: 58.5 (13.2) 2: 56.3 (11.2)	LBT, near; 1 (0.7) cm deviation LBT, far; 7 (6.6) cm deviation SCT, near; 0.9 (0.1) canceled/total SCT, far; 0.9 (0.0) canceled/total ACT; 0.8 (0.08) canceled/total	MoCA (≤22); N/A	NVisor HMD (1024 × 1280 pixels, 60 Hz refresh rate; 60° diagonal field of view); Attack3 Logitech Joystick	No head-tracking; Joystick responses	16
Peskine et al., 2011	9 (5/4)	50 (15)	HC; 9 (5/4)	50.6 (16.1)	BCT; 6 (4.9) omissions CBS^14^; 14 (7.7)	No screening	Unspecified HMD, electromagnetic sensor system	Head-tracking; mouse button responses	15

*The authors do not state which unit the score reflects.

*^1^Unilateral Spatial Neglect, ^2^Standard Deviation,^ 3^Right Hemisphere Stroke Patients without USN, ^4^Bells Cancellation Test, ^5^Apples Cancellation Test, ^6^Line Bisection Test, ^7^Montreal Cognitive Assessment, ^8^Healthy Controls, ^9^Letter Cancellation Test, ^10^Mini-Mental State Examination, ^11^Raven’s Colored Progressive Matrices, ^12^Wechsler’s Memory Scale, ^13^Star Cancellation Test, ^14^Catherine Bergego Scale, ^15^The Appraisal Tool for Cross-Sectional Studies (Downes et al., 2016).*

#### General Characteristics

Characteristics regarding USN participants, controls, conventional USN assessments, and cognitive screening tools are summarized in Table 2.

Table 2 shows the range in the number of included USN participants (9 to 16) and controls (9 to 20). The sampled age range was smaller in the USN groups (from M = 50, SD = 15 to M = 60.7 SD = 9.1), than the control groups (from M = 25.5, SD = 2.5 to M = 61, SD = 11.3). All studies except one (Aravind and Lamontagne, 2017) included a healthy control (HC) group. Kim et al. (2004) used HC groups with high and low computer experience. Five studies included a control group of right hemisphere stroke patients without USN (non-USN) (Kim et al., 2010; Aravind and Lamontagne, 2017; Ogourtsova et al., 2018a,b,c). Two studies used the same USN participants (Ogourtsova et al., 2018a,b). All included USN participants suffered from right hemisphere stroke and left USN.

#### Virtual Reality Characteristics

All studies used an HMD, although two failed to specify the model (Kim et al., 2010; Peskine et al., 2011). Four studies used an HMD with 1024 × 1280 pixels, and a 60° total diagonal field of view (Aravind and Lamontagne, 2017; Ogourtsova et al., 2018a,b,c). Three studies use an HMD with a refresh rate of 60 Hz (Ogourtsova et al., 2018a,b,c) while the remaining studies did not report on it (Kim et al., 2004, 2010; Peskine et al., 2011; Aravind and Lamontagne, 2017). Two studies tracked the position of the body and head as participants moved into the room (Aravind and Lamontagne, 2017; Ogourtsova et al., 2018b), while three studies only tracked the head (Kim et al., 2004, 2010; Peskine et al., 2011). Two studies employed no head-tracking (Ogourtsova et al., 2018a,c). Two studies tracked the head with three degrees of freedom (Kim et al., 2004, 2010), while the rest did not report on this. Regarding VR input controls, two studies used a joystick (Ogourtsova et al., 2018a,c) and two a computer mouse (Kim et al., 2010; Peskine et al., 2011). The remaining studies tracked the headset (Kim et al., 2004) or both the headset and body markers (Aravind and Lamontagne, 2017; Ogourtsova et al., 2018b). None of the studies used any measurements of presence or simulator sickness (summarized in Table 2).

#### Stimuli Characteristics

All studies primarily used visual stimuli, two included 2D sound cues (Kim et al., 2004, 2010), and one an auditory task (Aravind and Lamontagne, 2017). All studies used dynamic stimuli, meaning moving through virtual space. We considered all the tasks in the studies naturalistic, as tasks demands were related to ADL. Four studies applied an abstract virtual environment not resembling a naturalistic situation (Kim et al., 2004; Aravind and Lamontagne, 2017; Ogourtsova et al., 2018a,b). The remaining studies applied a naturalistic virtual environment, simulating real-life settings (summarized in Supplementary Material 3).

#### Virtual Reality Study Design and Outcomes

##### Detection and Navigation

The study of Kim et al. (2004) utilized a detection and visual scanning paradigm in which they placed the participants in front of a virtual branch road and measured their subjective midline as the deviation angle from the actual midline. The participants had to move a fixation cross to the location of a ball using head movements and maintain their fixation while the ball was moving. The location of the ball was not fully described but appeared to assess egocentric USN in peripersonal space. The study reported large effect sizes for all main outcomes between USN and HC groups (Outcome measures and main findings for all studies are reported in Supplementary Material 3).

In another detection task, Kim et al. (2010) utilized a street crossing design. Participants’ deviation angle was measured. They were steering an avatar (3D person) with a mouse in a traffic scene, through a third-person view. The avatar was placed at the curb, horizontally centered in front of a crosswalk at an intersection and crossing at the change of a traffic light. The participant had to keep the avatar safe by pressing a mouse button to stop an approaching car. If a car went undetected, visual cues in extrapersonal space and auditory cues were given. The authors reported that this task measured extrapersonal and egocentric USN and medium to large effect sizes for all main measures between USN and non-USN groups.

Another detection and a navigation task had participants navigate in a virtual city to locate swings in a park or at bus stops on both the left and right side in a first-person-view whilst moving forward through mouse clicks (Peskine et al., 2011). Seated in a swivel chair, participants could turn in order to change their point of view and the direction of movement. In this task, extrapersonal and egocentric USN were reported to be assessed. The study found large effect sizes for all main measures between the USN and HC groups.

The study of Ogourtsova et al. (2018a) used detection and a navigation task. Using a joystick seated participants had to navigate to a target 7 m away in VR presented either on the left, right side or in front of the participant inside their field of view. The navigation task had three different conditions: navigation to (1) a visible target, (2) a presented target that disappeared and had to be remembered as navigation began, and (3) a target that shifted location during navigation. The detection task had targets appearing at random positions in the field of view and required pushing a joystick button on detection of the target. This study reported measuring extrapersonal, egocentric USN. It found small to large effect sizes for mediolateral error for left-sided targets in remembered (2) and shifting conditions (3), and large effect sizes for detection times of targets regardless of position between USN and non-USN groups.

The study conducted by Ogourtsova et al. (2018b) used the same design without the detection task but used physical walking for navigation to measure extrapersonal, egocentric USN. Medium to large effect sizes were found for heading errors in several conditions: left and right targets in the visible (1) and remembered (2) condition between USN and HC/non-USN groups, the left target in remembered vs. visible conditions, and remembered vs. shifting (3) conditions within the USN group.

In the study of Ogourtsova et al. (2018c), they used another navigation and detection task, in a virtual supermarket. A symmetrical grocery shopping aisle with three shelves was placed 3 m in front of the participant. They were asked to locate a target cereal box and move toward it with a joystick in two conditions: a simple one, with only the target box appearing, and a complex one with the target box and distractor boxes. The target was located at eye level, but randomly appeared in five different locations on the left, right, and in the middle of the scene to measure extrapersonal, egocentric USN. Small to large effect sizes were found when comparing the complex and the simple condition within the USN group. Small to large effect sizes were found between USN participants and non-USN groups in the complex condition.

##### Dual Tasking

The study of Aravind and Lamontagne (2017) used an obstacle avoidance task, combined with a pitch-discrimination task. The study contained three conditions: (1) avoiding moving obstacles while walking, (2) a pitch-discrimination task, and (3) performing the pitch-discrimination task while avoiding obstacles. From the description of the placement of the obstacles, we inferred the study assessed extrapersonal, egocentric USN. Small to very large effect sizes were found between collision rates of USN and non-USN participants in all conditions, especially for contralesional obstacles. USN participants generally performed worse than HC on both pitch discrimination and obstacle avoidance while performing both tasks simultaneously.

#### The Relation of Virtual Reality Results to Conventional Unilateral Spatial Neglect Assessments

All but one study analyzed the correlation of scores from conventional USN assessments with VR measures (Peskine et al., 2011). Only Aravind and Lamontagne (2017) failed to find a significant correlation. Kim et al. (2004) reported a significant correlation between deviation angle and the Line Bisection and Letter Cancellation Tests. Kim et al. (2010) found a significant correlation between deviation angle and percent deviation from the Line Bisection Test (*r* = 0.63), but not for the main VR task performance. Correlations were found between reorientation strategies to the leftmost targets during shifting condition and Line Bisection Task in near (*r* = 0.29) as well as far space (*r* = 0.34) (Ogourtsova et al., 2018a) as did endpoint heading error during remembering condition (near: *r* = 0.42, far: *r* = 0.34) (Ogourtsova et al., 2018b). In case of the latter study, Star Cancellations Task in near (*r* = 0.34) and far space (*r* = 0.30) also correlated. Navigation time to target correlated weakly with all clinical USN measures, except the egocentric measure for the Apples cancellation test (*r* = 0.38–49) (Ogourtsova et al., 2018c).

Regarding potential false negatives of conventional measures and higher sensitivity of VR tasks than conventional measures, four studies found behavior indicative of USN in patients with a previous history of USN and non-USN patients. Three patients with a history of USN (out of 15), and four non-USN patients (out of 15) had significantly higher mediolateral displacement errors than the HC group (Ogourtsova et al., 2018a). Further, three patients with a history of USN (out of 15) and one non-USN participant (out of 15) performed worse than the HC group on endpoint heading error in the visible and remembered condition (Ogourtsova et al., 2018b). Ogourtsova et al. (2018c) found altered performance for three non-USN patients (out of 15), compared to the HC group when moving to the leftmost target. Peskine et al. (2011) found that two patients who did not test positive for USN and four patients with normal scores on the Bells Cancellation Task exhibited behavior indicative of USN in VR on left-to-right ratio on bus stops omitted.

### Virtual Reality Discussion

#### Summary of Findings

On lateralized measures, all included studies found significant differences in groups between USN patients and non-USN, HC, or both. They reported large effect sizes for performance on at least one contralesional measure compared to the control group (range: *g* = 0.8–2.94). The largest effect sizes were found in the more complex VR tasks involving multitasking, and when compared to HC groups. Only one study examined peripersonal, egocentric USN (Kim et al., 2004), whereas the remaining studies measured extrapersonal, egocentric USN (Kim et al., 2010; Peskine et al., 2011; Aravind and Lamontagne, 2017; Ogourtsova et al., 2018a,b,c). Four studies detected abnormal contralesional performances on VR measures in patients with non-USN (Peskine et al., 2011; Ogourtsova et al., 2018a,b,c). Five studies found significant, but low correlations between traditional assessments and VR performance (Kim et al., 2004, 2010; Ogourtsova et al., 2018a,b,c). Our quality assessment revealed methodological issues regarding the selection of patients, i.e., including patients with no current symptoms in the USN-group and not screening for cognitive deficits, and lack of piloting of the VR tools prior to the studies. The included studies appear to provide an improvement to the ecological validity of USN assessment over pen-and-paper methods. The outcome measures mostly used both results from the task and the process of completing it. In addition, all studies used naturalistic tasks with dynamic stimuli, although only three studies used naturalistic virtual environments (Kim et al., 2010; Peskine et al., 2011; Ogourtsova et al., 2018c).

No study differentiated between subtypes of USN or reported treatment effects of immersive VR tasks on USN. A summary and the authors’ interpretation of the results can be found in Table 3.

**TABLE 3:** Overview and authors’ interpretation of findings in VR studies.

		Task		
		Detection	Navigation	Dual tasking
Feature	Collision-rates *Rate of collision with obstacles in VR*		Aravind and Lamontagne, 2017	Aravind and Lamontagne, 2017
	Conventional assessment *Assessment tools e.g., BIT, CBS or cancellation tasks related to any VR measure*	Kim et al., 2004; Kim et al., 2010; Peskine et al., 2011	Peskine et al., 2011; Ogourtsova et al., 2018a,b,c	Aravind and Lamontagne, 2017
	Cue-dependency *Need for cues for detection*	Kim et al., 2004; Kim et al., 2010		
	Deviation angle *Subjective midline in VR*	Kim et al., 2004; Kim et al., 2010		
	Directional path deviation *Deviation to one side while moving*		Ogourtsova et al., 2018a,b,c	
	Individual profiling *Ability to assess USN on individual level*		Ogourtsova et al., 2018a,b,c	
	Omissions *Failure to detect stimuli*	Kim et al., 2010; Peskine et al., 2011		
	Subtypes *Ability to assess subtypes of USN e.g., allocentric USN*			
	Target, visible *Navigation to a visible target*		Ogourtsova et al., 2018a,b	
	Targets, disappearing *Navigation to a target that disappears while moving*		Ogourtsova et al., 2018a,b	
	Targets, shifting position *Navigation to a target that shifts position while moving*		Ogourtsova et al., 2018a,b	
	Time, Detection *Time to detect a target stimulus*	Kim et al., 2010; Ogourtsova et al., 2018a,c		
	Time, Scanning *Time spent scanning for a stimulus*	Kim et al., 2004		

*Overview of findings across VR studies. Except “Individual profiling” all findings are on group-level. Colors indicate level of evidence judged by the following criteria: (1) the number of studies able to detect and/or differentiate USN from non-USN or HC, (2) number of studies not able to do so and (3) the magnitude of the calculated Hedges g following Lenhard and Lenhard (2016) benchmarks (≥0.2 = small, ≥0.5 = medium, and ≥0.8, large). Green = Good evidence, one or more studies find large effect sizes. Yellow = Medium evidence, one or more studies find large or medium effect sizes but an equal number studies find no differences. Red = Poor evidence, no studies find any differences. Gray = in the present data this feature was not analyzed/measured.*

#### Detection of Unilateral Spatial Neglect Symptoms

Based on results and effects sizes from the included studies, the ability of immersive VR to detect USN symptoms seems promising. When examining effect sizes in relation to diagnostic validity of the assessment tool, a test can be considered an appropriate diagnostic marker if the effect size is larger than a *g* of 3, indicating that 5% or fewer of the relevant clinical group’s scores fall within the distribution of scores obtained by the control group (Zakzanis, 2001). Following this, while all studies found significant differences with large effect sizes between control groups and USN patients none were large enough to be considered a diagnostic marker of USN, although some came very close (*g* = 2.27–2.94) (Kim et al., 2004, 2010; Aravind and Lamontagne, 2017). Thus, these current studies could not reliably distinguish between groups of USN and control participants. In addition, detecting differential subtypes of USN on an individual level may be of great diagnostic importance for the planning of treatment. However, none of the included studies reported doing so.

Despite no studies finding effect sizes large enough to qualify as diagnostic markers in USN groups, several studies found patients in the non-USN group performing significantly different from the mean of HCs or the remaining non-USN group. This seems like preliminary evidence for the increased sensitivity of the VR instruments, compared to conventional methods.

There are indications of low concurrent validity, as five studies found significant, but low correlations between conventional and VR measures. However, given the limitations of the conventional assessments in terms of especially specificity and ecological validity, these correlations should not be interpreted as direct evidence against the suitability of the VR assessment methods.

#### Subtype Diagnostics

None of the included studies distinguished between USN subtypes, which may be problematic as some aspects of USN might not be differentiated or even detected. Subtype diagnostics may further improve prognostics on the USN patient’s functional independence and advance individual tailoring of treatment. However, information about different USN manifestations might be deduced from the studies. For instance, Ogourtsova et al. (2018b) measured both head orientation and locomotor mediolateral displacements and reported significant group differences in terms of mediolateral displacements, but not head orientation. This could be preliminary evidence for differentiation between different USN subtypes, as it hinted at normal head movement, but abnormal walking paths by USN patients, although no analysis was made on an individual level.

The studies did not specifically target bottom-up attention. Patients can compensate for an initial lateral attentional bias by redirecting their attention top down to the neglected side. This orienting is not assessed in any included study, even though measures during the first seconds of head orientation or deviation from paths could be tracked and analyzed.

#### Ecological Validity

##### Tasks, Measures, and Stimuli

Current studies of immersive VR discussed several traits for improving the ecological validity for USN assessment. Six studies leveraged the afforded three-dimensional interactions to assess extrapersonal USN, usually not covered by traditional methods. They generally used VR designs relying on situations not normally accessible for assessment, since navigation, street crossing, and obstacle avoidance are usually difficult to do in real life in a systematic, safe, and controlled fashion. Studying navigation abilities can be challenging for wheelchair-bound patients. However, in the designs of three studies joystick controls made it possible to study navigation in VR environments. Although the effect sizes were generally larger in the walking condition, the joystick studies found comparable and promising results, indicating that studies using joysticks could provide information about the navigation abilities when not walking (Ogourtsova et al., 2018a,b).

All studies used naturalistic tasks with demands similar to real life (e.g., navigation with or without obstacles or finding items). Multitasking paradigms are more sensitive to USN (Bonato, 2012), and the one study that utilized this, found some of the largest effect sizes (*g* = 2.27) in the complex multitasking condition (Aravind and Lamontagne, 2017). In addition, increasing the complexity of the stimuli yielded higher effect sizes (*g* = 0.48–2.27) (Aravind and Lamontagne, 2017; Ogourtsova et al., 2018c), providing evidence and justification for including such tasks in assessment.

All studies used naturalistic, dynamic stimuli, thus increasing the similarity to real-life tasks (verisimilitude) (Parsons, 2011). However, the measure with the highest effect size was deviation angle (*g* = 2.65–2.94) (Kim et al., 2004, 2010). This measure describes the patient’s subjective midline and is not part of the dynamic VR task, thus not possessing verisimilitude. Whether this task is able to predict behavior in real life (veridicality) has not been tested but could be examined by investigating correlations with ecologically valid assessment measures (e.g., CBS).

All included studies used outcome measures that quantified both the result and the process of completing a task, assessing for instance detection time and lateralization of navigation patterns. This should make it easier to detect abnormal ways of task completion, increasing sensitivity to USN (Joseph et al., 2014). The included tasks revealed displacements of walking trajectories from USN patients. Furthermore, these measures should be more sensitive to subtle differences due to the higher granularity of data in continuous variables such as degrees and time that would not reach levels of significance in categorical variables such as left/right cancelation ratios. Taken together, we hypothesize that the above features are part of the reasons why several studies (Peskine et al., 2011; Ogourtsova et al., 2018a,b,c) found deficits in VR tasks that were not found in or assessed by conventional tests.

##### Virtual Reality Technological Characteristics

An analysis of the VR characteristics revealed several suboptimal features related to immersion, which could reduce the feeling of presence, that the participant experiences. This is problematic because presence is considered one of the main mechanisms to improve the ecological validity of VR assessments and is a key factor in rendering the participants’ behavior more in line with real-life during dynamic 3D tasks (Parsons, 2011).

The only sensory modality sufficiently accommodated was the visual, as only three studies used audio stimuli, but in two of these, the audio-only served as cues. In addition, the few studies that reported field of view used an HMD with a narrow field of view (60°) diagonally, while the recent consumer HMDs provide a diagonal field of view of at least 110° (HTC, 2019; Oculus, 2019). There were also problems related to tracking, as only two studies tracked the whole body, and two studies did not use head tracking. Of the studies using head-tracking, only two studies reported on the degrees of freedom (DOF) of the tracking as three, out of six possible DOFs. Four studies used a joystick or a mouse button as their input controller that provided a weaker sense of presence than walking or haptic gloves providing visual and proprioceptive feedback (Bohil et al., 2011). The refresh rate of the visual scene in studies that disclosed this was 60 Hz, while the recent consumer HMDs feature refresh rates of 90 Hz (HTC, 2019; Oculus, 2019). However, the studies still facilitated high immersion from the HMDs used in all studies, which blocked out external visual stimuli, although no study blocked out the sound from the surrounding environment (but no study reported on distracting noise). Only the most recent studies reported on HMD resolution – with 1024 × 1280 pixels per eye equivalent to recent HMDs (HTC, 2019; Oculus, 2019). However, Kim et al. (2004) and Kim et al. (2010) reported “low graphics” regarding the display but did not disclose the resolution.

Specifically, the field of view, degrees of freedom, and refresh rate have all been found to correlate with self-reported presence with at least a medium-sized association (Cummings and Bailenson, 2016). Thus, the technological limitations in these studies may produce a lower sense of presence in VR than could be achieved using newer technology.

In general, the technology appears feasible for the patient group, as no study reported any adverse effects of using VR or mention any concerns for clinical use, e.g., in terms of hygiene. Low personal computer (PC) experience did not influence performance in VR tasks (Kim et al., 2004), in line with previous studies (Huygelier et al., 2019).

#### Treatment

None of the studies applied immersive VR to USN treatment, although the technology does seem appropriate and sensitive to central aspects of USN.

## Eye-Tracking Review

### Eye-Tracking Results

#### Study Selection

**FIGURE 2:**
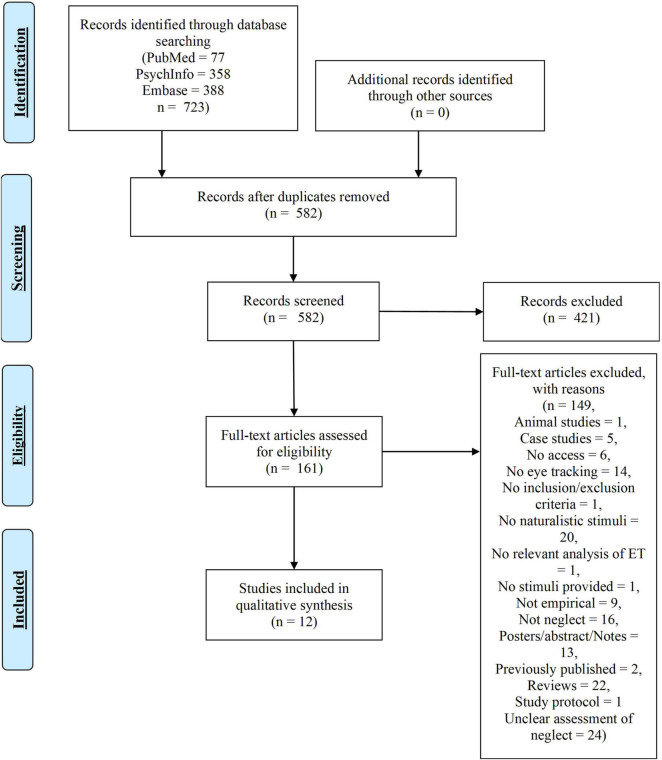
Eye-tracking (ET) search flow chart.

In total 723 records were identified. Removing duplicates left 582 records. Titles and abstracts were screened using the inclusion criteria listed in section 2.2, yielding 161 records for full-text eligibility screening. Twelve papers were included in the review (see Figure 2 for an overview).

#### Quality Assessment

Appraisal tool for cross-sectional studies (AXIS)-scores ranged from 10 to 16 (M = 13.71, SD = 1.8) (see Table 4). No studies justified their sample size; thus, potential risk of bias may be present regarding statistical power. All but one study used a selection process likely to include participants representative of the target population (Ohmatsu et al., 2019). Only two studies applied a screening of cognitive functioning (Leyland et al., 2017; Ohmatsu et al., 2019) but without a cut-off score and Ohmatsu et al. (2019) included patients scoring 0 on the Mini-Mental State Examination indicating a massive cognitive impairment. Thirteen USN patients seemed to be included in two different papers but with no explicit statement by the authors (Serino et al., 2006, 2007). Scores for each AXIS item are presented in Supplementary Material 4.

**TABLE 4:** Eye-tracking (ET) general characteristics.

Authors	USN^1^ participants *N* (male/female)	USN participants mean age (SD^2^)	Type of controls; *N* (male/female)	Control group Mean Age (SD)	Conventional USN assessment; Mean USN patient score (SD)	Cognitive screening (cut-off); Mean USN patient score (SD)	ET^3^ Technology	Head restrained, Y/N (type)	AXIS^4^-score/19
Cazzoli et al., 2016	8 w./VFD^5^ (6/2)	w./VFD: 56.63 (15)	VFD: 8 (4/4) Non-USN^6^: 8 (5/3) HC^7^: 8 (5/3)	VFD: 55 (8.54) Non-USN: 50.88 (14.76) HC: 60.67 (19.43)	BCT^8^: 1.38 (0.2) (difference in omissions) LBT^9^: 7.99%-point, (8.2%-point) CBS^10^: 9.63 (4.06)	None	iView X HED (SMI, SensoMotoric Instrumen, GmbH, Teltow, Germany), Sample rate: 50 Hz, Spatial resolution: < 0.1°, HM, right eye	N	15
Cazzoli et al., 2011	13 (7/4)	54.85 (8.65)	HC: 13 (6/5)	HC: 52.38 (7.03)	BCT: 4 (2.54) (left omissions, one missing datapoint) LBT: 12.19%-point (6.71%-point) (4 missing datapoints) Drawing: 1.23 (0.8) Reading omissions: 8 yes, 3 no, 2 missing PVT^11^: Omissions left:4.34 (3.68) 1 missing Omissions right:0.25 (0.56) 1 missing RT left: 4.35 (1.32), 1 missing RT, right: 1.57 (0.72),1 missing	None	Video-based, EyeLink (Sensomotoric Intstriments GmbH, Teltow, Germany), temporal resolution: 250 Hz, spatial resolution:0.01 degree, Compensates for head movements, Mounting unknown	Y (Chin rest)	14
Fellrath and Ptak, 2015	23 (16/7), 5 w./HEM^12^ (4/1)	64.4 (4) w./HEM 57.9 (9.3)	HEM: 10, (5/5), HC: 10 (4/6)	HEM: 48.9 (17.3) HC: 60.9 (8)	BCT: USN:10.5 (4.7) w./HEM: 14.6 (0.9) (left omissions) LCT^13^: USN: 17.9 (9.4), w./HEM: 20.3 (8.6) (left omissions) LBT: USN: 8.2%-point (10.9), w./HEM: 17.2%-point (11.3) SC^14^: USN: 7.9 (7.4), w./HEM: 0.2 (0.4) (missed words) Drawing: USN: 0.7 (0.9), w./HEM: 0.2 (0.4)	None	ETL-300, (ISCAN Inc., Woburn, MA, United States), sample rate: 120 Hz, resolution: 0.5 degrees, accuracy: 1 degree, Contact-free, right eye	N	14,5
Leyland et al., 2017	6, (NA)	64.67 (11.31)	Non-USN 6 (NA) HC: 9 (NA)	Non-USN: 73.20 (9.83) HC: 67.56 (9.95)	BIT^15^: 75.55 (8.46)	NART^16^ (NA), NA MMSE^17^ (NA), NA	Applied Science Laboratories E5000 eye-tracker, sample rate: 60 Hz, Head Mounted goggles, right eye.	Y (Chin rest)	10
Machner et al., 2012	19 (10/9)	70 (14)	Non-USN:14 (3/11) HC: 21 (NA)	Non-USN: 63 (19) HC: 69 (9)	MCT^18^: 6 (8) (Omissions: left-right) SC: 6 (8) (Omissions: left-right) LBT: 34%-point (28) FC: 3 (1) (N omissions) Reading: 65 (52) (Word omissions)	None	Red-X, (SensoMotoricInstruments, Teltow, Germany), Sample rate: 50Hz, Contact-free	Y (head stabilized)	16
Machner et al., 2018	Moderate: 12 (7/5) Severe: 12 (9/3)	Moderate: 68 (3) Severe: 69 (3)	Non-USN: 10 (3/7) HC: 11 (3/8)	Non-USN: 71 (3) HC: 69 (4)	CBS, moderate: 8 (1) CBS, severe: 24 (1) BCT, moderate: CoC^19^:0.32 (0.07) BCT, severe: CoC:0.77 (0.07) SC, moderate: CoC:0.3 (0.1) SC, severe. CoC:0.68 (0.1) LBT, moderate: 14.8 mm (5) LBT, severe 21.7 mm (3.2) Reading, moderate: 33 (13) (errors) Reading, severe: 68 (14) (errors) Copying, moderate: 2.7 (0.4) Copying, severe: 4 (0)	None	Red-X, (SensoMotoricInstruments, Teltow, Germany), Sample rate: 50 Hz, Contact-free	N	12
Ohmatsu et al., 2019	27 (NA)	74.5 (11.4)	Non-USN: 14 (NA) HC: 29 (9/20)	Non-USN: 65.8 (12.25) HC: 51.6 (18)	BIT: 92.8 (34.4) (inkl. 0-scores). CBS-O: 7.2 (8),1 missing CBS-S: 4 (5.35), 1 missing	MMSE (none) 20.34 (7.2) (inkl. 0-score)	PC Eye Go (Tobii Technology, Stockholm Sweden), Sample rate: 30 Hz Contact-free	N	12
Paladini et al., 2019	22 (13/9)	56.68 (9.49)	HC: 23 (11/12)	HC: 62.09 (17.66)	LBT: NA BCT, CoC: NA SCT, CoC: NA RSC^20^, CoC: NA	None	Infrared, T120 (Tobii Technology, Stockholm, Sweden), Sample rate: unknown, Contact-free	Y (Chin + head rest)	15
Primativo et al., 2015	10 (4/6)	68 (10.3)	Non-USN: 10 (6/4)	Non-USN: 71.2 (7.4)	LCT left: 36 (18.73) (omissions) LCT right: 15.6 (15.16) (omissions) LiCT^21^ left: 3.6 (3.92) (omissions) LiCT right: 1.2 (2.57) (omissions) WJA^22^ left: 8.1 (6.94) (unexpected) WJA right: 1.1 (1.66) (unexpected) Reading: 2.2 (2.4) (errors)	None	Eye Link 1000 (SR Research Ltd., Mississauga, Ontario, Canada), Sample rate: 500 Hz, Spatial resolution:0.04 degree, Contact-free monocular tracking	Y (head rest)	14
Ptak et al., 2009	7 (3/4)	64.4 (7.4)	Non-USN: 6 (4/2) HC: 18 (11/7)	Non-USN: 59.9 (10.3) HC: 37.6 (11)	BCT: 6.4 (4.2) left omissions TCT^23^; 12.3 (11.2) left omission LCT: 7.9 (6.7) left omissions LBT: 10%-point (6), right deviation Drawing: 1.3 (8)	None	HighSpeed (SMI, Germany), Sample rate: 240 Hz, Spatial resolution:0.3 degree, No further details	Y (chin rest)	15
Serino et al., 2006	USN experimental:16 (8/8) USN active control:8 (5/3)	USN experimental: 66.31 (9.73) USN active control: 67.75 (6.6)	None	NA	BIT-C exp: 98.5 (22.5) BIT.C con: 109.13 (39.1) BIT-B exp: 49 (16.1) BIT-B con: 52.8 (20.4)	None	Oculometer (Dr. Bouis Instruments, Germany), Spatial resolution: 5 min of arc, Sample rate: 500 Hz, Tracking left eye, No further details	Y (chin rest + head strap)	12
Serino et al., 2007	21 (11/10)	65.57 (12.25)	USN divided into groups based on adaption to PA^24^.	NA	BIT-B: 48.95 (15.3) BIT-C: 100.14 (22)	None	Oculometer (Dr. Bouis Instruments, Germany), Spatial resolution: 5 min of arc, Sample rate: 500 Hz, Tracking left eye, No further details	Y (chin rest + head strap)	15

*^1^Unilateral Spatial Neglect, ^2^Standard Deviation, ^3^Eye-tracking, ^4^The Appraisal Tool for Cross-Sectional Studies (Downes et al., 2016), ^5^Visual Field Deficit, ^6^Right Hemisphere Stroke Patients without USN, ^7^Healthy controls, ^8^Bells Cancellation Task, ^9^Line Bisection Test, ^10^Caterine Bergego Scale (Objective or Subjective scale), ^11^undescribed subtask from the Vienna Test System, ^12^Hemianopia, ^13^Letter Cancellation Task, ^14^Star Cancellation Task,^15^Behavioral Inattentional Task (Behavioral or Cognitive battery), ^16^National Adult Reading Test, ^17^Mini Mental State Examination, ^18^Mesulam Cancellation Task, ^19^Center of Cancellation, ^20^Random Shape Cancellation, ^21^Line Cancellation Task, ^22^Wundt-Jastrow Area Illusion, ^23^Inverted T Cancellation Task, ^24^Prismatic Adaption training.*

#### General Characteristics

Sample sizes ranged from 7 to 25 in patient groups and 6 to 29 in control groups. Age varied less in USN participants (M = 54.85, SD = 8.65 to M = 74.5, SD 11.4) compared to the control groups (M = 37.6, SD = 11 to M = 73.20, SD = 9.83). Three studies had no HC groups (Serino et al., 2006, 2007; Primativo et al., 2015). Five studies included a non-USN control group (Ptak et al., 2009; Machner et al., 2012, 2018; Cazzoli et al., 2016; Leyland et al., 2017), four studies used non-USN patients as controls – two with either visual field deficits of hemianopia (Fellrath and Ptak, 2015; Cazzoli et al., 2016) and two with right hemisphere lesions (Primativo et al., 2015; Ohmatsu et al., 2019). Furthermore, two studies used groups of USN patients either grouped on adaptiveness to treatment (Serino et al., 2007) or on whether they received an experimental or active control treatment (Serino et al., 2006). In one study, multiple experimental sub-studies were conducted on subgroups of USN patients grouped based on performances in an ET task.

All USN participants suffered from right hemisphere stroke or lesions and left USN (summarized in Table 4).

#### Eye-Tracking Characteristics

In further ET results, six studies used contact-free ET (Machner et al., 2012, 2018; Fellrath and Ptak, 2015; Primativo et al., 2015; Ohmatsu et al., 2019; Paladini et al., 2019), two head-mounted (Cazzoli et al., 2016; Leyland et al., 2017), and the remaining provided no description. Sampling rates ranged from 30 to 300 Hz. Five studies report precision (spatial resolution) albeit in different units ranging from.04 to.3 degrees (Ptak et al., 2009; Cazzoli et al., 2011, 2016; Fellrath and Ptak, 2015; Primativo et al., 2015), two studies reported using a spatial resolution of 5 min of arcsin (Serino et al., 2006, 2007) and the remaining studies provide no information. Eight studies used some form of head restraint (Serino et al., 2006, 2007; Ptak et al., 2009; Cazzoli et al., 2011; Machner et al., 2012; Primativo et al., 2015; Leyland et al., 2017; Paladini et al., 2019).

#### Eye-Tracking Conditions and Stimuli Characteristics

All studies but one (Leyland et al., 2017) assessed extrapersonal neglect as inferred from task descriptions and technical setups (see Supplementary Material 5). One study used both a naturalistic ET task and an experimental task with sparse analysis of the relationship between these (Machner et al., 2018). Two studies used ET as an effective measure of prism adaptation training (Serino et al., 2006, 2007) (summarized in Supplementary Material 5).

##### Static Free-View

Among the other studies, six used static photos of everyday scenes in free-view conditions. Three applied both original and mirrored versions to minimize bias of features in photos (Fellrath and Ptak, 2015; Ohmatsu et al., 2019; Paladini et al., 2019), one assumed symmetry in the photos (Ptak et al., 2009), one showed photos with different spatially distributed features (Machner et al., 2012) and one used a non-immersive VR traffic scene with cars and a road and did not consider the distribution of features (Cazzoli et al., 2016).

Furthermore, four studies analyzed features of pictures using saliency maps calculated from color, intensity, and orientation (Fellrath and Ptak, 2015; Paladini et al., 2019); brightness, color, static contrast, and dynamic contrast (Machner et al., 2012) or luminance, chromatic contrast, luminance contrast and edges (Ptak et al., 2009). One study only judged features qualitatively when including photos (Ohmatsu et al., 2019) and one study did not consider features (Cazzoli et al., 2016).

USN patients’ eye movements were attracted by less salient features in the right side of a photo compared to the left and one study found that luminance attracted USN patients’ gaze to their left hemifield (Ptak et al., 2009). One study found increased attraction to more salient features in the first 10 fixations in USN patients both with and without hemianopia (Fellrath and Ptak, 2015). In one study not controlling for scene features, USN patients showed a rightward bias exploring areas further to the right than non-USN patients with or without visual field deficits, but without differences in time spent or the distribution of eye movements across the scene (Cazzoli et al., 2016).

##### Dynamic Free-View

Two studies applied dynamic stimuli in a free-view-condition in either a non-immersive virtual environment of a virtual road with vehicles (Cazzoli et al., 2016) or a screen displaying video clips from nearby community areas (Machner et al., 2012). In a low-loaded condition virtual environment with few objects and no distractors, there were no differences in the distribution of fixations, but USN patients on average looked further right than non-USN and HC in early search, a bias present also in a high-loaded condition (Cazzoli et al., 2016). Further, USN patients with visual field deficits more often fixated on previously fixated areas in their left hemifield than non-USN with similar deficits. In free-viewing of video clips, USN patients on average looked further right than HC and non-USN (Machner et al., 2012). Low-loaded videos provoked a similar bias, which high-loaded videos increased further. USN patients’ gaze was more attracted by video features compared to random movement. Further, the dynamic movement was considered key in attracting their gaze. USN patients fixated on features with less movement to the right of their average fixation point, compared to the left of that point.

##### Static, Task-Based

Asked to search for either a paper clip among distractor items on a desk (Machner et al., 2018) or a naturalistic target (e.g., a leaf or a clock) in naturalistic photos (Cazzoli et al., 2011), moderate and severe USN patients detected fewer targets than HC and non-USN in their outer left hemifield, all patient groups were slower than HC and tempo and detection got worse as the severity of USN increased (Machner et al., 2018). They also found fewer objects in both upper and lower left quadrant than HC and GroupWise they found fewer targets in the lower than upper left quadrant in the first 10 s (Cazzoli et al., 2011). For USN patients the area in which their gaze mostly fell was narrower than for non-USN patients and HC (Machner et al., 2018).

##### Dynamic, Task-Based

When searching for targets in videoclips of the urban environment, USN patients looked at areas with more dynamic contrast on the right side compared to non-USN and HC and in general found fewer targets than HC since they seldom searched the left, although dynamic targets were more often found than static ones (Machner et al., 2012).

##### Drawn Stimuli

Two studies (Primativo et al., 2015; Leyland et al., 2017) presented patients with drawn images, which shares similarities with conventional tasks. In the cookie-theft image from the Boston Aphasia Battery, eye movements patterns from four out of ten USN patients deviated from those of HC (Primativo et al., 2015). Asked to copy or draw-along pairs of faces, the accuracy of left halves was poor during copying indicating allocentric neglect opposite during drawing-along, the accuracy of the right half of the left face increased, but the accuracy of the left face, in general, was worse indicating egocentric neglect (Leyland et al., 2017). In both conditions, an undescribed gaze distribution matched the drawing behaviors.

##### Reading Tasks

Using ET during the reading of letter-strings as an outcome of prismatic adaption training compared to general cognitive training (Serino et al., 2006) or improvement across sessions (Serino et al., 2007), showed that USN patients’ first saccade was further left after prismatic adaption training (Serino et al., 2006, 2007) and their ability to adapt to training during the first week was the better predictor of improvements (Serino et al., 2006). Also, the eye movements of USN patients were more spread out when reading letter-string and single-word-reading (Serino et al., 2007). USN patients with the deviating distribution of eye movements had more omissions and substitutions in left halves of both sentence and single-word-reading than other USN patients (Primativo et al., 2015).

##### Experimental Stimuli

In an experimental task with a dot appearing in one out of five horizontal or vertical positions for ten seconds, USN patients with the deviating distribution of eye movements were less accurate than other USN patients as well as HC both horizontally and vertically positions (Primativo et al., 2015). In a task with a square appearing on a screen in three conditions: with/without a fixation cross, or in the right side only, the same USN patients were less accurate and had smaller leftward saccades than other USN patients and HC, and an effect of side-of-target was found, but not elaborated on. All groups, including USN patients with/without deviating eye movement distributions, had lower accuracy for the left side compared to the right.

#### EM Measure Characteristics

##### First Saccade

Two studies analyzed the direction (Ptak et al., 2009; Cazzoli et al., 2011) and two studies analyzed the amplitude (Serino et al., 2006, 2007) of the first saccade. Findings showed that USN patients had a higher likelihood of a rightward-directed first saccade (Ptak et al., 2009; Cazzoli et al., 2011). The study by Ptak and colleagues predominantly relied on descriptive statistics therefore it is unclear if findings are statistically significant. Findings from studies on prismatic adaption training suggested that the first saccade may be directed more leftward after two weeks of training (Serino et al., 2006, 2007) and it may be present after 1 and 3 months (Serino et al., 2007). The latter two studies included very few participants (*N* = 8 and *N* = 21) and not all underwent ET evaluation or were available for follow-up.

##### Saccade Amplitude

Unilateral spatial neglect (USN) patients had shorter saccades in different directions (Ptak et al., 2009; Machner et al., 2012; Fellrath and Ptak, 2015) and in both directions (Machner et al., 2012). USN patients’ regardless of having hemianopia or not exhibited smaller saccades than hemianopia patients (Fellrath and Ptak, 2015). Only one study found smaller saccade amplitude in four USN patients with deviating eye movement distribution compared to both non-USN and other USN patients (Primativo et al., 2015).

##### Saccade Numbers and Distribution

Among other studies, two found no differences in the number of saccades (Ptak et al., 2009; Fellrath and Ptak, 2015), and a single study found that USN patients produced fewer saccades in their left hemifield compared to non-USN patients and HC (Machner et al., 2012).

##### Saccade Directions

In further results, two studies found no differences in the percentage of leftward directed saccades in USN patients free-viewing or searching photos and videos (Machner et al., 2012; Fellrath and Ptak, 2015) and one study found no difference in numbers of saccades in either direction (Ptak et al., 2009). In two studies, USN patients’ leftward saccades were of lesser amplitude compared to rightward saccades for both naturalistic (Machner et al., 2012) and experimental material (Primativo et al., 2015).

##### Trajectory

Regarding trajectory, one study found that USN patients’ cumulated trajectories of eye movements were shorter than for non-USN patients and HC, suggested to be a result of the USN patients producing saccades of lesser amplitude (Ptak et al., 2009).

#### EM Measure Characteristics – Fixations

##### Spatial Distribution of Fixations

Among other studies, six had reported distributions of fixations in four quadrants (Cazzoli et al., 2011), six horizontal columns (Cazzoli et al., 2016), the proportional number of fixations across the horizontal axis (Ptak et al., 2009; Ohmatsu et al., 2019) median position of fixations on the x-axis (Machner et al., 2012), as a correlation between several fixations and x-coordinates, or as the mean location of fixations on the horizontal axis (Paladini et al., 2019). Cazzoli et al. (2011) found a skewed distribution of fixation in only the early phase of visual search, while Cazzoli et al. (2016) only found it during visual search in a dynamic task in USN patients with visual field deficits when comparing them to non-USN patients and HCs. Three studies applying static stimuli found a rightward skewness for USN patients compared to non-USN and HC (Ptak et al., 2009; Ohmatsu et al., 2019; Paladini et al., 2019) a single study reported a similar skewness in non-USN compared to HC (Ohmatsu et al., 2019). One study applied both dynamic and static material and found a rightward skewness in both free-view and search conditions for USN patients compared to non-USN and HC (Machner et al., 2012).

##### Number of Fixations

No differences in several fixations were found between USN and non-USN patients (Primativo et al., 2015), USN patients with or without hemianopia, nor between USN patients with/without hemianopia and non-USN patients with hemianopia nor HCs (Fellrath and Ptak, 2015). During visual searches, USN patients had more fixations prior to detection, but the number did not differ across target placements (Cazzoli et al., 2011). A single study reported a lower mean number of fixations in USN patients compared to non-USN patients and HCs when free-viewing photos though without any thorough analysis (Ptak et al., 2009).

##### Re-fixations and Perseveration

Analysis of fixations within a Euclidian distance of ≤32 pixels of a previous fixation (re-fixations) and fixations landing within the same Euclidian distance of a re-fixation (perseverations), showed that USN patients were more likely to have a first re-fixation in their right hemifield and had predominantly right hemifield re-fixations opposed to HC, who did so in the left hemifield (Paladini et al., 2019). These differences were found after 10 seconds of search and USN patients further deviated from HC by re-fixating and first-time fixating d on more salient areas in the left hemifield. USN patients made more perseverations than HCs in both hemifields.

##### Duration of Fixations

Prolonged fixations in the right hemifield were not USN specific. While both USN and non-USN patients exhibited these (Primativo et al., 2015) non-USN patients with hemianopia and HCs (Ptak et al., 2009; Fellrath and Ptak, 2015) showed the same bias in free-viewing photos. When comparing performances on static and dynamic stimuli, shorter fixations in static compared to dynamic stimuli were not USN specific whereas USN patients had prolonged fixations compared to non-USN and HC and USN patients’ fixations lasted longer in the right compared to the left hemifield (Machner et al., 2012).

#### EM Measure Characteristics – Gaze

In further results, five studies reported the spatial distribution of gaze points (Serino et al., 2006, 2007; Cazzoli et al., 2016; Leyland et al., 2017; Machner et al., 2018). Cazzoli et al. (2016) found a rightward bias in USN with visual field deficits during static and dynamic conditions compared to controls. One study found a rightward bias when comparing severe and moderate USN patients (assessed on CBS), but not moderate USN and non-USN though all USN groups were further right than HC (Machner et al., 2018). Severe USN patients looked in a narrower area than non-USN and HC. In drawing of faces, USN patients spent less time on the left halves of faces when drawing along and on the leftmost face when copying (Leyland et al., 2017). After prismatic adaption training USN patients spent more time in their left hemifield (Serino et al., 2006, 2007).

#### Eye Movements Related to Conventional Neglect Measures

The study of Machner et al. (2018) reported a positive relation between CBS and median gaze position during visual search (*r* = 0.705). Further, increasingly leftward placed the first saccade after prismatic adaptation training correlates with improvement on the total Behavioral Inattentional Task-score indicating less USN (*r* = −0.61) though proportional fixation distribution did not correlate with this (Serino et al., 2006). Behavioral Inattentional Task-scores correlated with the difference in gaze position in different search conditions for USN patients only (*r* = −0.58). For both USN and non-USN patients’ correlations with the initial (5 ms) (*r* = −0.54) and later search (450 ms) (*r* = −0.56) were found; all revealed a negative relationship with the increasingly rightward placement of gaze (positive value) leading to worse Behavioral Inattentional Task-scores (Ohmatsu et al., 2019). In the same study observational CBS score correlated with both initial search (*r* = 0.4), later search (*r* = 0.44), and gaze shift and velocity in the left (*r* = −0.41) and right (*r* = −0.54) hemifield for both types of patients.

### Eye-Tracking Discussion

#### Summary of Findings

##### Neglect Behavior Across Types of Stimuli and Task Condition

In general, USN patients were attracted to more salient features in static photos when looking to the left side and luminance was most successful in attracting gaze (Ptak et al., 2009; Fellrath and Ptak, 2015; Ohmatsu et al., 2019; Paladini et al., 2019). In dynamic videos, USN patients showed a rightward bias when the complexity of features and movement increased and dynamic features attracted patients’ gaze better than other features in video-stimuli (Machner et al., 2012; Cazzoli et al., 2016). USN manifests itself as a preference for searching the right hemifield in both static and dynamic stimuli with luminance and complexity of stimuli as central features.

In visual search of static stimuli, USN patients found fewer targets in the outer left hemifield (Machner et al., 2018), found fewest in their lower left quadrant, and found fewer targets in the upper half their total hemifield (Cazzoli et al., 2011). During dynamic stimuli, fewer targets were found in the left hemifield since patients depend heavily on guidance by features if they are to search out this hemifield (Machner et al., 2012). Hence, the preference for searching the right side is also present during dynamic stimuli and it is relying on stimuli features.

Patients with severe USN can be separated from other USN patients on visual search (Machner et al., 2018). In verbal descriptions of drawings, only a few USN patients’ search patterns deviate from those of non-USN patients’ as do USN patients’ eye movements differ on reading tasks and they have smaller, slower, and less accurate eye movements in experimental tasks (Primativo et al., 2015). Further, drawing accuracy seems to follow the distribution of eye movements and both may distinguish between both ego- and allocentric USN (Leyland et al., 2017). Thus, USN patients can be distinguished from either non-USN or other subtypes of USN through visual search and drawing tasks.

All studies used naturalistic stimuli and found significant differences comparing USN and non-USN/HC although there were major differences in effect sizes (Range: *g* = 0.54–8.74).

No studies used ET for the treatment of USN. A summary and the authors’ interpretation of the results can be found in Table 5.

**TABLE 5:** Overview and authors’ interpretation of findings in ET studies.

		Task					
		Free-view, Static stimuli	Free-view, Dynamic stimuli	Drawing	Search, Static stimuli	Search, Dynamic stimuli	Reading
Feature	Conventional assessment *Assessment tools e.g., BIT, CBS or Cancellation tasks related to any ET measure*	Ptak et al., 2009; Ohmatsu et al., 2019; Paladini et al., 2019			Machner et al., 2018		Serino et al., 2007
	Fixations, Duration *Duration in ms of fixations.*	Machner et al., 2012; Fellrath and Ptak, 2015; Primativo et al., 2015	Machner et al., 2012				
	Fixations, Distribution *Distributions in either columns, quadrants or on axes*	Ptak et al., 2009; Machner et al., 2012; Fellrath and Ptak, 2015; Cazzoli et al., 2016; Ohmatsu et al., 2019; Paladini et al., 2019	Machner et al., 2012; Cazzoli et al., 2016		Cazzoli et al., 2011		
	Fixations, *N Total number of fixations*	Ptak et al., 2009; Fellrath and Ptak, 2015; Primativo et al., 2015			Cazzoli et al., 2011		
	Fixations, Refixating *Fixating in the same area one or more times*	Paladini et al., 2019					
	Gaze *Considering all types of eye movements*	Cazzoli et al., 2016; Ohmatsu et al., 2019	Cazzoli et al., 2016	Leyland et al., 2017	Machner et al., 2018		Serino et al., 2006, 2007
	Individual profiling *Ability to assess USN on individual level*	Primativo et al., 2015					
	Saccades, Amplitude *Amplitude of saccades in one or more directions*	Ptak et al., 2009; Machner et al., 2012; Fellrath and Ptak, 2015; Primativo et al., 2015	Machner et al., 2012			Machner et al., 2012	Serino et al., 2006, 2007
	Saccades, First *Direction, amplitude or hemifield of first saccade*	Ptak et al., 2009			Ptak et al., 2009; Cazzoli et al., 2011		Serino et al., 2006, 2007
	Saccades, Direction *Number or ratio of saccades directed left/right*	Ptak et al., 2009; Fellrath and Ptak, 2015			Cazzoli et al., 2011	Machner et al., 2012	Serino et al., 2007
	Saccades, *N Total number of saccades*	Ptak et al., 2009; Machner et al., 2012; Fellrath and Ptak, 2015	Machner et al., 2012				
	Saccades, Trajectory *Sum of distance of all saccades*	Ptak et al., 2009					
	Subtypes *Ability to assess subtypes of USN e.g., allocentric USN*			Leyland et al., 2017			

*Static stimuli = stimuli without any movement e.g., still images. Dynamic stimuli = stimuli with movements e.g., video clips. Except “Individual profiling” all findings are on group-level. Colors indicate level of evidence judged by the following criteria: (1) the number of studies able to detect and/or differentiate USN form non-USN or HC, (2) number of studies not able to do so and (3) the magnitude of the calculated Hedges g following Lenhard and Lenhard (2016) benchmarks (≥0.2 = small, ≥0.5 = medium, and ≥0.8, large). Green = Good evidence, one or more studies find large effect sizes. Yellow = Medium evidence, one or more studies find large or medium effect sizes but an equal number studies find no differences. Red = Poor evidence, no studies find any differences. Gray = in the present data this feature was not analyzed/measured.*

##### Neglect Behavior in Eye Movements

Amplitude seems the best marker of USN when considering saccades. The saccade amplitudes, in general, were smaller in USN patients in all directions (Ptak et al., 2009; Machner et al., 2012; Fellrath and Ptak, 2015) and smallest in USN patients with deviant eye movement distributions (Primativo et al., 2015) resulting in the shorter total trajectory of eye movements (Ptak et al., 2009). A rightward direction of the first saccade was USN specific (Ptak et al., 2009; Cazzoli et al., 2011) but not the number of directions of saccades in general (Ptak et al., 2009; Machner et al., 2012; Fellrath and Ptak, 2015).

A rightward bias in fixations was present for USN patients in both static (Ptak et al., 2009; Ohmatsu et al., 2019; Paladini et al., 2019) and dynamic stimuli (Cazzoli et al., 2016) and was present in early visual search tasks (Cazzoli et al., 2011). The number of fixations does not seem to differ between USN patients (Fellrath and Ptak, 2015; Primativo et al., 2015), but they have more fixations prior to target detection in the lower left quadrant during visual search (Cazzoli et al., 2011). Though, USN patients show an opposite pattern to controls with predominantly re-fixations in their right hemifield (Paladini et al., 2019). USN patients have prolonged fixations in their right hemifield for both static (Ptak et al., 2009; Fellrath and Ptak, 2015) and dynamic stimuli (Machner et al., 2012). Therefore, the lack of evenly distributed fixations when searching static and dynamic stimuli could be thought of as an indicator of USN with a tendency to re-fixating and to have prolonged fixations in the right hemifield as underlying measures of an impaired ability to self-guidance of visual attention.

USN patients seem to have a rightward biased distribution of their gaze (Leyland et al., 2017; Machner et al., 2018), though it is unclear whether there is a difference between types of tasks (Cazzoli et al., 2016) or not (Machner et al., 2012). Interestingly, time spent looking at stimuli in drawing tasks fits allocentric and egocentric behavior in drawing accuracy (Leyland et al., 2017), and alterations of gaze following prismatic adaption training seemingly led to better scores on the Behavioral Inattentional Task (Serino et al., 2007). Also, gaze distinguishes between different severities of neglect as well as differentiating non-USN patients from HCs (Machner et al., 2018). Thus, gaze may be able to differentiate both subtypes and severities of USN from each other and HCs, as well as provide knowledge of the underlying attentional mechanisms in USN.

#### Detection of Unilateral Spatial Neglect

ET can apparently detect USN using saccade amplitude (Ptak et al., 2009; Machner et al., 2012; Fellrath and Ptak, 2015; Primativo et al., 2015), fixations distribution (Ptak et al., 2009; Cazzoli et al., 2011, 2016; Ohmatsu et al., 2019; Paladini et al., 2019), fixations prior to target detection (Cazzoli et al., 2011) re-fixations (Paladini et al., 2019) and distribution of gaze (Ptak et al., 2009; Machner et al., 2012, 2018; Fellrath and Ptak, 2015; Cazzoli et al., 2016; Leyland et al., 2017), as well as differentiate between severities (Machner et al., 2018), and sub-types of USN (Leyland et al., 2017) and provide analysis of the individual patient (Primativo et al., 2015). Yet, the link between ET measures and specific cognitive impairments of USN is unclear although suggestions exist (Hill et al., 2015; Cameirão et al., 2016). Since significant differences in ET measures such as gaze differed across task conditions (Cazzoli et al., 2016) and the number of saccades in the left hemifield did not (Machner et al., 2012), it seems equally relevant to consider if different ET measures across different task conditions could reflect different attentional impairments. Following the diagnostic validity guideline by Zakzanis (2001) of *g* > 3, six studies (Cazzoli et al., 2011; Machner et al., 2012, 2018; Fellrath and Ptak, 2015; Leyland et al., 2017; Ohmatsu et al., 2019) found sufficient effect sizes (*g* = 3.02–8.74). Thus, ET appears to have a very promising ability to reliably distinguish between groups of USN and control participants and serve as a reliable diagnostic marker.

The literature provided some evidence in terms of concurrent validity, as a further leftward first saccade in USN patients correlated with better Behavioral Inattentional Task-scores (*r* = −0.61) (Serino et al., 2006) and scores correlated with greater shifts in initial search (*r* = −0.544) and the following search (*r* = −0.561) (Ohmatsu et al., 2019).

#### Subtypes Diagnostics

Though no studies analyzed eye movement on the individual subtype diagnostics level, features such as distribution of gaze during free-viewing of drawing/images (Primativo et al., 2015), distribution of eye movements during drawing tasks (Leyland et al., 2017), and distribution of fixations during different tasks (Cazzoli et al., 2011, 2016; Machner et al., 2012, 2018) have the possibility of assessing the individual eye-movement characteristic.

#### Ecological Validity

##### Tasks, Measures, and Stimuli

Only studies applying stimuli related to a recognizable everyday setting were included to reflect high verisimilitude (Parsons, 2016). Only two studies used both naturalistic and experimental stimuli (Primativo et al., 2015; Machner et al., 2018), whereas the Posner task used by Machner et al. (2018) was not compared to the naturalistic task. USN patients seem to have difficulties focusing their attention on all parts of naturalistic stimuli similar to scenes in everyday life, i.e., composed of different features such as color, luminance, and edges that are not evenly distributed (Ptak et al., 2009; Fellrath and Ptak, 2015; Paladini et al., 2019). Thus, verisimilitude is achievable in ET designs.

Considering veridicality ET has promising potentials since median x-position in visual search (*r* = 0.705) (Machner et al., 2018) and shift in USN patients’ gaze in initial (0.5 s, *r* = 0.398) to remaining search (1.5 s, *r* = 0.439) (Ohmatsu et al., 2019) all correlated with the CBS (Bartolomeo, 2014). Further, it is hard to imagine if ET measures should differ greatly between ET setups using naturalistic stimuli and everyday settings due to the degree of verisimilitude. Thus, veridicality is secured using ET since the biases in eye movement distributions can be considered similar to the distributions in patients’ every day which underlies their impaired ADL-function.

##### Eye-Tracking Technological Characteristics

No clear preference for neither mounting technology nor software properties was found across studies, partly due to four studies with insufficient specifications of the used technology. Sampling rates ranged from 30 to 300 Hz and spatial resolution ranged from.04 to.3 degrees if reported. Thus, the technological properties of each included study varied, raising the concern that a lower granularity of data may have overlooked possible USN-specific behaviors. Further, not all types of eye movements were classified identically e.g., only three out of seven studies defined saccades, defining them using different criteria (see Supplementary Material 5). All but one study (lacking a definition) defined fixations as stable eye positions over at least 80–100 ms (see Supplementary Material 5). Therefore, comparisons across studies are not straightforward and contradictory findings may be due to differences in definitions.

A more central question is whether participants had their heads restrained. In six studies, heads were restrained using either head or chin rests (Ptak et al., 2009; Cazzoli et al., 2011; Machner et al., 2012; Primativo et al., 2015; Leyland et al., 2017; Paladini et al., 2019) and two studies used head straps (Serino et al., 2006, 2007). In an everyday setting, heads are not restrained, and patients’ gaze can be moved using both oculomotor and neck muscles by turning the head, leaving little if any insight into attentional bias from either oculomotor or a general motor deficit. Some ET solutions can account for and correct for head-movement (Cazzoli et al., 2011) paving the way to understanding the underlying mechanisms of USN behavior in everyday settings.

#### Treatment

None of the studies applied ET in USN treatment, although ET seems to measure underlying mechanisms of USN and therefore yield future clinical potential.

## Discussion and Future Perspectives

This review suggests that (1) several features of VR and ET seem suitable for detecting USN on a group level, such as multitasking in complex VR environments and detailed eye-movement analysis. Nevertheless, (2) across studies only a few of the features have been applied to individual subtype diagnostics, though several features show promise in this regard. The ecological validity of USN assessment (3) may be increased by assessing USN in everyday situations such as navigation and street crossing (verisimilitude), though only a few studies systematically tested the prognostic validity of VR and ET assessment on everyday behavior (veridicality). Finally, this review did not discover (4) any study that has utilized VR or ET technologies for the treatment of USN up until the 26th of February 2020 when the search was completed.

### Assessment of Unilateral Spatial Neglect With Virtual Reality and Eye-Tracking

The existing literature provides evidence that assessment of USN using immersive VR systems may provide novel and sensitive measures of spatial attention that are not readily accessible in conventional tests or from clinical observations. Likewise, the use of ET in the assessment of USN may provide more precise diagnostics of visual attention and oculomotor performance. However, the existing literature primarily analyzed VR-ET data on a group level overlooking possibilities of individual USN subtypes diagnostics, such as the presence of normal eye movement distributions in some but not all USN patients (Primativo et al., 2015). Therefore, an implicit assumption of homogeneity in the USN syndrome is not as clinically applicable as a heterogeneous view, with the possibility of multiple individual symptoms. An immersive VR-ET combination may provide new possibilities of more nuanced individual USN subtype and differential diagnostics, which in turn will facilitate an individually tailored and specific treatment approach.

#### Conventional Tests

Conventional tests often suffer from low ecological validity, ceiling effects, and inadequate ability to differentiate USN subtypes (Ting et al., 2011). VR-ET assessments can avoid some of these shortcomings by presenting naturalistic environments, continuously monitoring attentional behaviors on a multimodal and multidimensional level, while recording spatial and temporal data in high resolution (Joseph et al., 2014), and assessing the underlying attentional guidance of behavior through eye-tracking (Ptak et al., 2009). Proof-of-concept of VR-ET-based assessment of USN may even be tested on healthy subjects treated with inhibitory repetitive transcranial magnetic stimulation (Schwab et al., 2021).

VR-ET systems can replicate conventional USN tests, e.g., cancelation and line-bisection, thus, providing more fine-grained analysis of the attentional processes during the tasks, such as the field of view, distribution of gaze, and response time. Novel VR-based visual search and cancellation tests reveal a high correlation with conventional USN tests (Knobel et al., 2020, 2021) and may improve our understanding of the field of perception (FOP) and field of regard (FOR) in stroke patients with and without USN (Kim et al., 2021).

Virtual reality (VR)-ET may help improve sensitivity and specificity by including more complex stimulus arrays, simultaneous stimuli, and multitasking paradigms (Bonato, 2012) in VR, e.g., by occupying top-down executive attention by a central discriminatory task, while simultaneously testing bottom-up spatial attention (Blini et al., 2016). Double dissociation between performance in static and dynamic tests may more reliably be revealed in controlled VR-based environments (Spreij et al., 2020).

Nevertheless, ecological validity may be increased by the use of naturalistic scenes such as the baking tray test (Tham, 1996; Fordell et al., 2011), grocery shopping (Ogourtsova et al., 2018c), street-crossing (Kim et al., 2010; Navarro et al., 2013; Cazzoli et al., 2016), or even a driving simulator (van Kessel et al., 2013), which all presumably share more similarities with ADL.

#### Neglect Subtype Diagnostics

The many facets of USN subtypes (e.g., sensory/motor, allo-/egocentric, extra-/peri-/personal) call for subtype-specific assessments (Buxbaum et al., 2004; Kerkhoff and Schenk, 2012; Rode et al., 2017). In a virtual environment, lateralization of USN (horizontal/vertical/radial) can be assessed by taking different frames of reference into account, such as allocentric (object-centered) and egocentric (body-centered) with different bodily midlines (trunk, head, and eyes). Hence, differences in inattention across body/object-midlines not readily assessable in conventional tasks can be analyzed in high spatial and temporal resolution.

In VR-ET egocentric (body-based) neglect can be detected by responses (e.g., omission errors and reaction times) in relation to the movement of the hands (controller), head (HMD), and eyes (ET) (Hougaard et al., 2021). Assessments of allocentric (object-based) neglect e.g., using drawings (Leyland et al., 2017) can be replicated and further developed into a variety of task types in VR-ET, where spatial attention to both halves of objects can be tracked. More ranges of space such as personal (bodily), peripersonal (within arm’s reach) and extrapersonal space (beyond arm’s reach) can be assessed by presenting stimuli across them (Yasuda et al., 2020).

Sensory neglect may be assessed by the use of multimodal stimuli (visual, tactile, auditory) and extinction to simultaneous stimuli. Visual neglect could be assessed by replicating conventional tasks (Tham, 1996; Fordell et al., 2011; Bonato, 2012) or introducing discriminatory tasks such as grayscale gradient or chimeric faces (Mattingley et al., 1993, 2004) whilst tracking eye movements.

In VR-ET sensory input and motor output can be altered in ways that are not possible in conventional testing, e.g., mirroring of hand movement for disentangling of sensory and motor USN (Na et al., 1998), or constraining the visual field for disentangling hemianopia and visual neglect (Beschin and Facchin, 2016). Furthermore, the attentional guidance of gaze is assessable (Machner et al., 2018), as is amplitude, direction, and distribution of different eye movements (Ptak et al., 2009).

### Treatment of Unilateral Spatial Neglect With Virtual Reality and Eye-Tracking

The scientific literature has described a multitude of therapeutic approaches addressing USN (e.g., Kerkhoff and Schenk, 2012; Bowen et al., 2013; Azouvi et al., 2017). Conceptually, treatment approaches are often categorized as top-down or bottom-up approaches (e.g., Bowen et al., 2013). Bottom-up approaches are interventions directed at the impairment without requiring awareness of the deficit. Top-down approaches are interventions that encourage awareness of the disability and the use of compensatory strategies. These treatment approaches may be implemented in VR-ET in different training game scenarios. Table 6 presents some of the most common treatment approaches and how they can be implemented in VR and ET. Some feasibility and usability studies (Huygelier et al., 2020; Morse et al., 2020; Chen and Krch, 2021), proof-of-concept studies (Wilf et al., 2020), protocols for validation in healthy adults (Cho et al., 2020) and controlled treatment studies (Bourgeois et al., 2021b; Choi et al., 2021), mostly focusing on implementation of prism adaptation training in VR, has been published after the literature search for this review was completed.

**TABLE 6:** Conventional and potential VR-ET treatment approaches.

	Conventional treatment	VR and ET implementation
Prismatic shift	The most commonly researched bottom-up treatment approach is prism adaptation training (PAT; e.g., Frassinetti et al., 2002). Patients wear prismatic goggles (typically 10 degrees Fresnel prisms) shifting the visual field into the non-neglected direction causing misalignment between the visual and motor system. The brain rapidly adapts to this shift, and after removal of the prismatic goggles this adaptation results in an aftereffect where a motor dislocation into the neglected direction is shortly present. By repeating PAT several times over the course of days and weeks a more permanent treatment effect can be observed.	In VR, prismatic shift can be obtained by manipulation of the alignment of the real arm and the virtual arm (Ramos et al., 2019; Wilf et al., 2020; Bourgeois et al., 2021a,b). By gradually introducing an offset of the virtual arm into the non-neglected hemispace, the patient is forced to reach increasingly further into the neglected hemispace to reach targets of the VR training game. The training game could be implemented as a simple whack-a-mole or balloon popping game or even a first-person shooter.
Mirror therapy	Mirror therapy is commonly used to train hemiparesis but has been documented to also alleviate severity of spatial neglect (e.g., Thieme et al., 2013; Pandian et al., 2014). Mirroring the use of the unaffected arm to the contralateral hemispace creates the illusion that the affected arm is moving as well. Traditionally mirror therapy is applied by placing a mirror at the patients’ midsagittal plane, with the reflecting side of the mirror to the unaffected arm.	Mirroring of arm movements can convincingly be implemented in VR simply by transposing the movement of the unaffected arm to both the unaffected and affected arm in VR. This can even be done with and without mirroring of the x-axis for different types of bimanual tasks. Mirroring of the x-axis would reflect real mirror behavior in traditional mirror therapy but transposing the movement of the unaffected arm to the affected arm without mirroring of the x-axis would allow for bimanual task with a fixed distance between the arms, such as balloon popping or dual-gun first-person-shooter games.
Motor augmentation	Augmentation or exaggeration of movement is commonly used in commercially available treatment options such as arm robots. Augmentation of movement makes it possible for hemiparetic patients even with limited range of movement (e.g., small rotation of wrist) to participate in treatment games with the affected arm. Individually adjusted exaggeration of small hand movements to larger in-game movements increases the motivation of the patients to engage in treatment games.	Augmentation of movements are easily implemented in VR treatment and can be gradually adjusted to the capability of the individual patient or with the progress of training. Augmentation of movement could be applied to different 3D VR versions of classic arcade type games with simple movements such as ‘break out’ and ‘space invaders’.
Motor constraint	Constraint-induced motor therapy (CIMT) forces the use of the affected arm by applying a glove or mitt to the non-affected hand. The theory behind CIMT is based on the concept of learned-non-use and forced-use of the affected arm. The treatment effect of CIMT on upper limb hemiparesis has been well documented (Corbetta, 2014), but treatment effect on spatial neglect has also been shown (Wu et al., 2013).	Motor constraint is easily implemented in VR simply by solely rendering the affected arm perhaps in combination with motor augmentation if need be. VR motor constraint could be a self-rehabilitation supplement to traditional CIMT as high intensity is needed to establish treatment effects.
Visual constraint	Half-field eye-patching or visual constraint is a sensory counterpart of motor constraint, as the patient is forced to use the affected visual field by patching the unaffected visual field. The constraining of the unaffected visual field is believed to trigger the so-called Sprague effect by which the contralateral superior colliculus is inhibited and hemispheric balance restored (Sprague, 1966). This effect has even been shown to disentangle visual USN and hemianopia in some patients (Beschin and Facchin, 2016). Visual constraint by using half-field eye-patching has been shown to have positive treatment effects on spatial neglect (Smania et al., 2013).	Visual constraint can be implemented in VR by patching out half of the visual field or by placing the patient next to a virtual wall, hence constraining the unaffected visual field in relation to head or body midline. This therapeutic strategy can be applied to a multitude of VR games and simultaneously combined with other therapeutic approaches.
Visual Scanning Training	Visual Scanning Therapy also known as the lighthouse strategy sometimes combined with anchoring is among the most commonly used top-down therapeutic approaches (Niemeier et al., 2001). Visual scanning therapy is a compensatory treatment approach by which the patient is taught to scan the visual field from left to right as if their visual focus were the light beam of a lighthouse. A distinct visual cue (anchor) can be placed in the far most left of the visual field to notify the patient that they have reached the starting point.	These techniques can be implemented in a VR training game teaching patient to voluntarily explore the entire visual field. The ‘light beam’ of the lighthouse could be visualized relative to the head movement (HMD) or the eye movement inside the HMD and the position of the ‘anchor’ relative to the midline could adapt continuously to the capability of the patients’ performance. Literally, the game could be implemented as a lighthouse scenario guiding ships at sea, or training safe street crossings (Kim et al., 2010) and driving simulator (van Kessel et al., 2013).
Optokinetic stimulation	Optokinetic stimulation or smooth pursuit exploits the phenomenon that if one is placed in front of a screen that fills the entire field of view with objects moving from the right to the left, one gets the illusion that the body rotates to the right. One will usually respond by correcting for this illusion by re-orient to the left, which has been exploit in the treatment of neglect with good results (Kerkhoff et al., 2006, 2013). However, it seems to be an important prerequisite for the treatment effect is that the patient makes accompanying movements with the eyes, so-called smooth pursuit.	Optokinetic stimulation can be implemented in VR by different street-crossing and diving simulator scenarios, though cyber sickness may present a limitation of these. Diagnostics of eye movement by use of ET may be important in targeting optokinetic stimulation to the individual patients and establishing that the correct treatment techniques (i.e., smooth pursuit) and treatment effects.
Sustained attention training (SAT)	Sustained attention training is a bottom-up method that takes advantage of the fact that attention consists of at least two systems: A non-spatial alerting and a spatial orienting system. The theory is that activating the alerting system with external stimuli one can also increase the activity in the damaged spatial orientation system (Robertson et al., 1995). Commercial computerized training systems are available where the patient must respond as quickly as possible by reacting to particular targets and not to distractors (Degutis and Van Vleet, 2010).	Non-spatial sustained attention training paradigms can be implemented in VR and be designed for immersive and engaging gameplays, such as simple whack-a-mole or first person shooter with targets and distractors.
Cueing and feedback	Emotionally salient stimuli have been shown to improve visual search patterns when the targets expressed happy or fearful sensations, as opposed to their neutral equivalents (Klinke et al., 2015). Similarly, improved performance has been documented when patients were rewarded (monetary or points) for detecting leftward targets (Lucas et al., 2013). Visual but not auditory-verbal feedback seem to increase aftereffects following virtual prism adaptation (Bourgeois et al., 2021a).	Uni- or multimodal visual, auditory and tactile cueing (e.g., controller vibration or tactile gloves) and performance feedback could be systematically implemented in VR and used to enhance the treatment effect in combination with other treatment methods (e.g., Huygelier et al., 2020). Emotionally salient stimuli could be presented as a whack-a-mole type game smashing bees or flies and rewards could be presented as in game and between game performance high-scores.

Conventional treatment methods are time-consuming and resource-intensive to achieve sufficient treatment effects. Implementing treatment approaches in VR-ET will allow for rapid modifications and combinations of approaches. Treatment effects may in turn be increased by tailoring and continually adjusting the treatment to the needs and capabilities of the individual patients. Treatment programs should leverage adaptive algorithms capable of spawning the next target at the border of the individual patients’ attentional capacity (Knoche et al., 2017) relative to the patients’ current locus of attention. Treatment games should be encouraging in order to increase motivation (effort) and treatment intensity (gamification), i.e., provide in-game feedback on performance and between-game feedback on progress and support goal setting (Hougaard and Knoche, 2019). Implementation of individualized treatment in VR-ET training games may in near future make patients able to participate in self-training and telerehabilitation, which could increase the intensity of treatment (duration).

Apart from tracking hand, head, and eye movements, limb movements could be tracked through additional trackers, e.g., elbows and shoulders. A therapist-avatar could be presented in the virtual environment for patient-therapist interplay by adding a tracker to a therapist tablet-interface. Most systems are primarily based on visual and motor modalities (Bohil et al., 2011). However, auditory and haptic modalities should be added for multimodal feedback and interaction with the virtual environment.

### Future Research

We argue for untapped potentials in using combined VR-ET for assessment and treatment of USN and assessment of USN subtypes and differential diagnostics to inform individualized treatment choices. Likewise, we believe that the specificity, intensity, and efficacy of USN treatment could be improved by developing VR-ET systems, which allow for tailored treatment approaches, single or combined, to the individual patient-specific USN symptoms. These systems should be established by theory and empirically driven research based on existing knowledge from conventional tests and treatment approaches. Such VR-ET systems could provide the basis for future self- and telerehabilitation.

The present review focused solely on immersive technologies, as these are becoming increasingly affordable and usable for the general public and allow for clinical implementation. VR-ET hardware will inevitably be carried forward by technological improvements in off-the-shelf hardware, such as increased field of view, screen resolution, refresh rates of VR HMDs, and in-HMD eye-tracking devices with improved 2D/3D accuracy and sampling rates. This underlines the importance of establishing golden standards in VR-ET USN assessment and treatment software that can be swiftly and effortlessly be ported to off-the-shelf hardware.

We would like to encourage the scientific and clinical community in neurorehabilitation to collaborate on such VR-ET-based assessment and treatment programs in an open-source environment (e.g., GitHub or the openrehab.org initiative). By doing so, we can ensure that any progress made in developing, programming, and understanding the complex interplay between USN and interaction with technology can be shared across cultural, financial, and institutional boundaries.

### Limitations

A limitation of this review is the lack of independent raters regarding the inclusion and exclusion criteria. This could potentially be a source of bias in the selection of articles. However, the review provides full transparency in the inclusion criteria and the selection of articles, thus it should be completely reproducible.

In addition, due to the heterogeneity of the study methods and data structure, we were unable to perform a meta-analysis. In addition, several studies suffered from low quality assessment, which affects the strength of the conclusions. Lastly, VR and ET have had significant technological advances during the inclusion period, which could affect the generalizability of the review results.

## Data Availability Statement

The original contributions presented in the study are included in the article/Supplementary Material, further inquiries can be directed to the corresponding author.

## Author Contributions

AK and KV performed the database searches, selection and reviewed the studies, and were also the primary contributors to the methods, results, and discussions in the individual review sections. AS and HK assisted in writing the review sections. LE was the primary contributor to the overall introduction and discussion section. All authors contributed to further revisions of the manuscript.

## Conflict of Interest

The authors declare that the research was conducted in the absence of any commercial or financial relationships that could be construed as a potential conflict of interest.

## Publisher’s Note

All claims expressed in this article are solely those of the authors and do not necessarily represent those of their affiliated organizations, or those of the publisher, the editors and the reviewers. Any product that may be evaluated in this article, or claim that may be made by its manufacturer, is not guaranteed or endorsed by the publisher.
