# Supplementary material for: Virtual Reality and Eye-Tracking Assessment, and Treatment of Unilateral Spatial Neglect: Systematic Review and Future Prospects
Source: Front Psychol. 2022 Mar 22;13:787382. doi: 10.3389/fpsyg.2022.787382 (PMC8982678; doi:10.3389/fpsyg.2022.787382)
Supplement: Supplementary file 1 [file Table_1.docx]

# Supplementary Material 1

| **Supplementary Table 1**  *Overview over facets in systematic search* | | |
| --- | --- | --- |
| **Facet 1** | **Facet 2** | **Facet 3** |
| Unilateral spatial neglect | Eyetracking | Virtual Reality |

| **Supplementary table 2**  *Overview over Facets and Hits in Pubmed* | | |
| --- | --- | --- |
| **Facet 1** | | |
| *No.* | *String* | *Hits* |
| #1 | "unilateral neglect" OR "hemispatial neglect" OR "spatial neglect" OR "hemineglect" OR "hemi inattention" OR "hemi-inattention" OR "visual neglect" OR "auditory neglect" OR "tactile neglect" OR "allocentric neglect" OR "egocentric neglect" OR "sensory neglect" OR "motor neglect" OR "perceptual neglect" OR "hemi agnosia" OR "hemispatial agnosia" OR "unilateral agnosia" OR "neglect dyslexia" OR "representational neglect" OR "personal neglect" OR "peripersonal neglect" OR "extrapersonal neglect" OR "ipsilateral neglect" OR "vertical neglect" OR "radial neglect" OR "extinction to simultaneously stimuli" OR "motor extinction" OR "visual extinction" OR "auditory extinction" OR "tactile extinction" OR “neglect patient*” OR usn OR “visuospatial neglect” | 4190 |
| #2 | 1[Title/Abstract] | 3704 |
| #3 | perceptual disorders [MeSH Terms] | 27897 |
| #4 | "child neglect" OR (neglect AND child*) | 7665 |
| #5 | 2 OR 3 NOT 4 | 1615 |
| **Facet 2** | | |
| *No.* | *String* | *Hits* |
| #1 | eye tracking OR eye movements OR Saccades OR gaze movement* OR eye camera OR eye fixation* OR smooth pursuit | 62428 |
| #2 | 1[Title/Abstract] | 26674 |
| #3 | "Eye Movements"[Mesh] | 46738* |
| #4 | "Eye Movement Measurements"[Mesh] | 12372* |
| #5 | 3 NOT 4 | 41728* |
| #6 | 3 OR 4 | 54100* |
| #7 | 6 AND 2 | 18874 |
| **Facet 3** | | |
| *No.* | *String* | *Hits* |
| #1 | "virtual reality" OR "VR" OR "HMD" OR “Head- Mounted Display” OR CAVE OR “Cave Automatic Virtual Environment” OR “Virtual Environment" | 29218 |
| #2 | 1[Title/Abstract] | 20914 |
| #3 | “Virtual Reality”[Mesh] | 1524 |
| #4 | 2 OR 3 | 21370 |
| *Note:* *“Eye Movement Meassurements” [MeSH] was previously indexed under “Eye Movements” [Mesh] (1966-2006) albeit a quick runthrough of results in search 5 revealed studies applying eye tracking. Hence, the operator OR were used in search #6. | | |

| **Supplementary Table 3**  *Overview over Facets and Hits in PsycInfo* | | |
| --- | --- | --- |
| **Facet 1** | | |
| *No.* | *String* | *Hits* |
| #1 | **Any Field**:  "unilateral neglect" OR  "hemispatial neglect" OR  "spatial neglect" OR "hemineglect" OR  "hemi inattention" OR  "hemi-inattention" OR  "visual neglect" OR  "auditory neglect" OR "tactile neglect" OR  "allocentric neglect" OR  "egocentric neglect" OR  "sensory neglect" OR "motor neglect" OR "perceptual neglect" OR  "hemi agnosia" OR "hemispatial agnosia" OR  "neglect dyslexia" OR  "representational neglect" OR "personal neglect" OR "peripersonal neglect" OR  "extrapersonal neglect" OR  "ipsilateral neglect" OR  "vertical neglect" OR  "radial neglect" OR "motor extinction" OR  "visual extinction" OR  "auditory extinction" OR "tactile extinction" OR "neglect patient*" OR  usn OR  "visuospatial neglect" | 4097 |
| #2 | **Title:** #1 OR **Abstract:** #1 | 3067 |
| #3 | **Index Terms**: Sensory neglect | 2161 |
| #4 | 2 OR 3 | 3508 |
| #5 | **Index Terms:** (Child Neglect) OR (Antisocial Behavior) | 14783 |
| #6 | **Abstract:** (Children OR Child) | 584258 |
| #7 | 3 NOT 4 NOT 5 | 3489 |
| #8 | 6 AND **Peer-Reviewed Journals Only** | 3113 |
| **Facet 2** | | |
| *No.* | *String* | *Hits* |
| #1 | **Any Field:** “eye tracking” OR “eye movements” OR saccades OR “gaze movement*” OR “eye camera” OR “eye fixation*” OR “smooth pursuit” | 32563 |
| # | **Title:** #1 OR **Abstract:** #1 | 20067 |
| #2 | **Index Terms:** Eye Movements | 16939 |
| #3 | 1 AND 2 | 11781 |
| #4 | 3 AND **Peer-Reviewed Journals Only** | 10326 |
| **Facet 3** | | |
| *No.* | *String* | *Hits* |
| #1 | **Any Field:** "virtual reality" OR "VR" OR "HMD" OR “Head- Mounted Display” OR CAVE OR “Cave Automatic Virtual Environment” OR “Virtual Environment" **Peer-Reviewed Journals Only** | 12148 |
| #2 | **Title:** #1 OR **Abstract:** #1 | 7095 |
| #3 | **Index Terms:** Virtual Reality **Peer-Reviewed Journals Only** | 6519 |
| #4 | 2 OR 3 | 9776 |

| **Supplementary Table 4**  *Overview over Facets and Hits in Embase* | | |
| --- | --- | --- |
| **Facet 1** | | |
| *No.* | *String* | *Hits* |
| #1 | "unilateral neglect" OR "hemispatial neglect" OR "spatial neglect" OR "hemineglect" OR "hemi inattention" OR "hemi-inattention" OR "visual neglect" OR "auditory neglect" OR "tactile neglect" OR "allocentric neglect" OR "egocentric neglect" OR "sensory neglect" OR "motor neglect" OR "perceptual neglect" OR "hemi agnosia" OR "hemispatial agnosia" OR "unilateral agnosia" OR "neglect dyslexia" OR "representational neglect" OR "personal neglect" OR "peripersonal neglect" OR "extrapersonal neglect" OR "ipsilateral neglect" OR "vertical neglect" OR "radial neglect" OR "extinction to simultaneously stimuli" OR "motor extinction" OR "visual extinction" OR "auditory extinction" OR "tactile extinction" OR “neglect patient*” | 4645 |
| #2 | 1:ab,ti | 4405 |
| #3 | 'hemispatial neglect'/exp OR 'visual deprivation'/exp OR 'unilateral neglect'/exp OR 'visuospatial neglect'/exp OR 'spatial neglect'/exp OR 'hemineglect'/exp OR 'neglect dyslexia'/exp OR 'personal space'/exp OR 'peripersonal space'/exp OR 'visual extinction'/exp | 2897 |
| #4 | 2 OR 3 | 6371 |
| #5 | ‘antisocial behavior’/exp | 183747 |
| #6 | 4 NOT 5 | 5397 |
| **Facet 2** | | |
| *No.* | *String* | *Hits* |
| #1 | 'eye tracking' OR 'eye movements' OR saccades OR 'gaze movement*' OR 'eye camera' OR 'eye fixation*' OR 'smooth pursuit' | 43855 |
| #2 | 1:ab,ti | 30751 |
| #3 | 'eye movement'/exp OR 'eye movement monitor'/exp^a^ | 55611 |
| #4 | 2 OR 3 | 61391 |
| **Facet 3** | | |
| *No.* | *String* | *Hits* |
| #1 | "virtual reality" OR "VR" OR "HMD" OR “Head- Mounted Display” OR CAVE OR “Cave Automatic Virtual Environment” OR “Virtual Environment" | 76033 |
| #2 | 1:ab,ti | 26873 |
| #3 | ‘virtual reality’/exp | 16320 |
| #4 | 'virtual reality system'/exp | 44 |
| #5 | 'virtual reality simulator'/exp | 288 |
| #6 | 3 AND 4 AND 5 | 16,524 |
| #7 | 2 OR 6 | 34,446 |
| *Note:* ^a^'eye movement'/exp withholds both saccadic and smooth pursuit eyemovements and eye tracking, The term: eye movement measurement was not used since it emphasizes oculography not eye tracking. | | |
