# Supplementary material for: Virtual Reality and Eye-Tracking Assessment, and Treatment of Unilateral Spatial Neglect: Systematic Review and Future Prospects
Source: Front Psychol. 2022 Mar 22;13:787382. doi: 10.3389/fpsyg.2022.787382 (PMC8982678; doi:10.3389/fpsyg.2022.787382)
Supplement: Supplementary file 2 [file Table_2.docx]

**Supplementary Material 2**

**Supplementary Table 5**

*AXIS scores for VR review*

| **Authors** | **1** | **2** | **3** | **4** | **5** | **6** | **7** | **8** | **9** | **10** | **11** | **12** | **13** | **15** | **16** | **17** | **18** | **19** | **20** | **Score /19** |
| --- | --- | --- | --- | --- | --- | --- | --- | --- | --- | --- | --- | --- | --- | --- | --- | --- | --- | --- | --- | --- |
| Aravind and Lamontagne (2017) | 1 | 1 | 0 | 1 | 1 | 1 | 1 | 1 | 0 | 1 | 1 | 1 | 1 | 1 | 1 | 1 | 0 | 1 | 1 | 16 |
| Kim et al. (2004) | 1 | 1 | 0 | 1 | 0 | 0 | 1 | 1 | 0 | 1 | 1 | 0 | 1 | 1 | 0 | 0 | 1 | 1 | 1 | 12 |
| Kim et al. (2010) | 1 | 1 | 0 | 1 | 1 | 1 | 1 | 1 | 0 | 1 | 1 | 0 | 1 | 1 | 1 | 1 | 1 | 1 | 1 | 16 |
| Ogourtsova, Archambault, and Lamontagne (2018a) | 1 | 1 | 0 | 1 | 1 | 0 | 1 | 1 | 0 | 1 | 1 | 1 | 1 | 1 | 1 | 1 | 1 | 1 | 1 | 16 |
| Ogourtsova, Archambault, and Lamontagne (2018b) | 1 | 1 | 1 | 1 | 1 | 0 | 1 | 1 | 0 | 1 | 1 | 1 | 1 | 1 | 1 | 1 | 1 | 1 | 1 | 17 |
| Ogourtsova, Archambault, Sangani, and Lamontagne (2018) | 1 | 1 | 0 | 1 | 1 | 0 | 1 | 1 | 0 | 1 | 1 | 1 | 1 | 1 | 1 | 1 | 1 | 1 | 1 | 16 |
| Peskine et al. (2011) | 1 | 1 | 0 | 1 | 1 | 0 | 1 | 1 | 0 | 1 | 1 | 1 | 1 | 1 | 1 | 1 | 0 | 1 | 1 | 15 |
| % | 7/7 (100%) | 7/7 (100%) | 1/7 (14%) | 7/7 (100%) | 6/7 (86%) | 2/7 (29%) | 7/7 (100%) | 7/7 (100%) | 0/7 (0 %) | 7/7 (100%) | 7/7 (100%) | 5/7 (71%) | 7/7 (100%) | 7/7 (100%) | 6/7 (86%) | 6/7 (86%) | 5/7 (71%) | 7/7 (100%) | 7/7 (100%) | 15.4 (*SD*= 1.62) |
| *Note:* 1. Were the aims/objectives of the study clear? 2. Was the study design appropriate for the stated aim(s)? 3. Was the sample size justified? 4. Was the target/reference population clearly defined? (Is it clear who the research was about?) 5. Was the sample frame taken from an appropriate population base so that it closely represented the target/reference population under investigation? 6. Was the selection process likely to select subjects/participants that were representative of the target/reference population under investigation? 7. Were measures undertaken to address and categorize non-responders? 8. Were the risk factor and outcome variables measured appropriate to the aims of the study? 9. Were the risk factor and outcome variables measured correctly using instruments/measurements that had been trialed, piloted or published previously? 10. Is it clear what was used to determined statistical significance and/or precision estimates? (e.g., p values, CIs) 11. Were the methods (including statistical methods) sufficiently described to enable them to be repeated? 12. Were the basic data adequately described? 13. Does the response rate raise concerns about non-response bias? 15. Were the results internally consistent? 16. Were the results for the analyses described in the methods, presented? 17. Were the authors’ discussions and conclusions justified by the results? 18. Were the limitations of the study discussed? 19. Were there any funding sources or conflicts of interest that may affect the authors’ interpretation of the results? 20. Was ethical approval or consent of participants attained? (Downes, Brennan, Williams, & Dean, 2016) | | | | | | | | | | | | | | | | | | | | |
