# Supplementary material for: Virtual Reality and Eye-Tracking Assessment, and Treatment of Unilateral Spatial Neglect: Systematic Review and Future Prospects
Source: Front Psychol. 2022 Mar 22;13:787382. doi: 10.3389/fpsyg.2022.787382 (PMC8982678; doi:10.3389/fpsyg.2022.787382)
Supplement: Supplementary file 3 [file Table_3.docx]

| **Supplementary Material 3**  **Supplementary Table 6**  *VR study design, outcome measures and results* | | | | | |
| --- | --- | --- | --- | --- | --- |
| **Authors** | **VR^1^ Study Design** | **VR Stimuli** | **Subtype of USN^2^ Measured** | **Main VR Measures** | **Main Finding (Effect size)** |
| Aravind and Lamontagne (2017) | *Dual tasking; Obstacle avoidance*  Obstacle avoidance, pitch-discrimination task and a combination of both. | Visual, dynamic stimuli; abstract VE^3^; naturalistic task; visual stimulus with auditory task | Extrapersonal; egocentric  (inferred) | Collision rates (%); Minimum distance from obstacle (m); Onset of avoidance strategies (sec); pitch-discrimination error rates (%) | USN participants had higher collision rates especially for contralesional obstacles, on the walking condition (*g* = .14), the simple dual task (*g* = 1.36) and the complex dual task condition (*g* = 2.27) and had higher dual tasking cost than the non-USN^4^ group. Across conditions, USN participants tended to deviate to the ipsilesional side. |
| Kim et al. (2004) | *Detection; Visual scanning*  Participants had to move a fixation cross to the location of a ball and then follow the ball as it moved. | Visual stimulus with auditory cues; dynamic stimuli; poor graphics; abstract VE | Peripersonal; egocentric (inferred) | Deviation angle; No-attention time; Scanning time; Number of cues; Failure rate; The ratio of right and left scan time | Significant difference between USN and control groups on Deviation angle (HC^4^1: *g* = 2.94; HC2: *g* = 2.7); No-attention time (HC1: *g* = 1.07; HC2: *g* = 1.04); Scanning time (HC1: *g* = 1.47; HC2: *g* = 1.44); Number of cues (HC1&2: *g*=1.21); Failure rate ((HC1&2: *g* = .81); The ratio of right and left scan time (HC1: *g* = 1.07; HC2: *g* = .93).  Significant difference within USN group between performance in Right and left side of space in Scanning time (*g* = .92) and Failure rate (*g* = .8). |
| Kim et al. (2010) | *Street crossing; Detection*  As an avatar crossed at a crosswalk, the participant had to press a mouse button to stop an approaching car. | Visual stimulus with auditory cues; dynamic stimuli; naturalistic VE and task, but poor graphics; | Extrapersonal; egocentric | Deviation angle (°); Reaction time (sec); Left-to-right reaction time ratio; Left/right visual and auditory cue rates (%); Failure rates left and right (%) | USN group had significantly different Deviation angle (*g* = 2.65), Left-to-right reaction time ratio (*g* = 1.25), Left visual (*g* = 1.55) and Auditory cue rates (*g* = 1.82), and Left failure rates (*g* = .98) compared to the non-USN^5^ group.  Within the USN group, there was a significant difference between Left to Right reaction time ratio (*g* = .48), Left visual (*g* = 1.23) and Auditory cue rates (*g* = 1.51), and the left failure rate (*g* = .98). |
| Ogourtsova et al. (2018a) | *Detection; Navigation*  Detection of targets appearing at random positions.  Navigation with joystick to a target that were either visible, disappeared as navigation began or shifted location. | Visual, dynamic stimuli; abstract VE; naturalistic task; | Extrapersonal; egocentric | Endpoint mediolateral displacement error (lateral difference in meters between the target and the participant after moving forward 5 m); Direction of trajectory deviation; Onset of reorientation strategy for the shifting condition (sec); Detection time (sec) | USN group had greater mediolateral error to the leftmost targets in remembered (non-USN: *g* = .24, HC: *g* = .91) and shifting (non-USN: *g* = .54, HC: *g* = .78) conditions, lengthier onset of reorientation strategies (N/A) and slower detection times (-30° non-USN: *g* = .91, HC: *g* = 1.34; -15° non-USN: *g* = 1.22, HC: *g* = 1.7) compared to the control groups. |
| Ogourtsova et al. (2018b) | *Navigation*  Walking to a target that were either visible, disappeared as navigation began or shifted location. | Visual, dynamic stimuli; abstract VE; naturalistic task | Extrapersonal; egocentric | Endpoint heading error (difference in degrees between the ideal position and the subjects’ position in relation to the target at 5 m displacement); Endpoint mediolateral displacement (m); Head orientation (°); Endpoint direction; Onset of reorientation strategy (sec) | USN group had greater endpoint mediolateral displacement (N/A) and heading errors in actual (-15° non-USN: *g* = 1.67; HC: *g* = 1.39) and remembered (-15° non-USN: *g* = 1.54; HC: *g* = 1.42) condition and delayed onset of reorientation (N/A) for left and right targets compared to the control groups. Within the USN group there were larger heading errors for the left target in remembered vs. visible conditions (*g* *=* 1.28) and remembered vs. shifting conditions (*g =* .77). |
| Ogourtsova et al. (2018c) | *Detection; Navigation*  Detection of a cereal box with and without distractors and subsequent navigation towards the box. | Visual, dynamic stimuli; naturalistic VE and task | Extrapersonal; egocentric | Detection time (sec); Maximal mediolateral deviation from ideal path to target (m); Navigation time to target (sec) | USN group had significantly longer detection times for left and middle targets in the complex condition (N/A) and larger mediolateral deviations (-40° non-USN: *g* = .48) and longer navigation time (-40° non-USN: *g* = 1.02) in the complex condition for the left most target compared to the non-USN group. Within the USN group in the complex condition compared to the simple condition, larger mediolateral deviations for left and middle targets (-40°: *g* = .36; -20°: *g* = .29) and longer navigation times for the most eccentric left target (-40°: *g* = .75) were found. |
| Peskine et al. (2011) | *Detection; Navigation*  Participants navigated a virtual city to locate swings in a park or bus stops on both sides. | visual, dynamic stimuli; naturalistic VE and task | Extrapersonal; egocentric | Amount of bus stops omitted; Left-to-right ratio of omissions; Detection of swings | Significant difference between USN and HC group on Amount of bus stops omitted (*g* = 1.44); Left-to-right ratio of omissions (*g* = 1.1); and Detection of swings (N/A). |
| *Note:* ^1^Virtual Reality; ^2^Unilateral Spatial Neglect; ^3^Virtual environment, ^4^Healthy Control group, ^5^Right Hemisphere Stroke Patients without USN | | | | | |
