# Supplementary material for: Virtual Reality and Eye-Tracking Assessment, and Treatment of Unilateral Spatial Neglect: Systematic Review and Future Prospects
Source: Front Psychol. 2022 Mar 22;13:787382. doi: 10.3389/fpsyg.2022.787382 (PMC8982678; doi:10.3389/fpsyg.2022.787382)
Supplement: Supplementary file 5 [file Table_5.docx]

**Supplementary Material 5**

| **Supplementary Table 8**  *ET study design, outcome measures, and results* | | | | | |
| --- | --- | --- | --- | --- | --- |
| **Authors** | **ET^1^ task** | **EM^2^ Tracked, Type (definition)** | **Subtype of USN^3^ measured^b^** | **EM Measure** | **Main findings^a^** |
| Cazzoli et al. (2016) | Free-viewing a virtual traffic scene with two-way road, pedestrian-crossing, buildings and trees.  *Static condition*: Without traffic  *Dynamic condition*: Cars from both sides | Fixations (>100 ms)  Gaze (NA) | Extrapersonal | CFD^4^ %-wise, six columns of 30 degrees  Mean x-axis gaze position during first second of search. | USN patients w. VFD^5^ had a mean gaze position in early search further to the right compared to non-USN^6^ with VFD (*g* = 1.2) non-USN without VFD (*g* = 0.75) and HC^7^ (*g* = 1.28) in static condition.  USN patients w. VFD had a mean gaze position in early search further to the right compared to non-USN with VFD (*g* = 0.65) non-USN without VFD (*g* = 0.77) and HC (*g* = 0.70) in dynamic condition.  All groups had significantly different mean gaze positions across conditions.  No differences in CFD distribution in static condition was found.  In the outer left column USN patients had significantly lower CFD compared to USN with VFD (*g* = 2.76) and HC (*g* = 1.26). No differences in remaining columns. |
| Cazzoli et al. (2011) | Visual search in 32 colored everyday photos, searching for a naturalistic target (either: a clock, a leaf, a pear or a screw). Target in one of 4 positions in a quadrant of photo. | Fixations (>100ms)  Saccades (NA) | Extrapersonal | CFD %-wise, four quadrant (upper,lower,right,left).  N fixations until detection  Direction of first saccade (to one of the quadrants). | USN made fewer fixations in both the lower left compared to the lower right (*g* = 2.57) and the upper left compared to the upper right (*g* = 2.25). No differences in HC.  USN patients’ first saccade was more often directed to the upper right than the upper left quadrant (*g* = 3.29) and more often to the lower right than lower left quadrant (*g* = 2.24). HC showed opposite behavior with preference for the upper left (*g* = -1.13) and the lower left (*g* = -1.1) quadrant.  USN produced more fixations prior to detecting a target for targets in the lower left quadrant than HC (*g* = 1.1). |
| Fellrath & Ptak (2015) | Free-viewing roughly symmetrical photos of natural scenes for 8 seconds. Saliency maps for each photo was computed from: orientation, luminance contrast/intensity and color. | Fixations (>80 ms)  Saccades (>1^0^ amp.) | Extrapersonal | Amp. of saccades  N saccades in each direction (left/right)  Duration of fixations  N fixations in each hemifield (left/right)  CFD %-wise, four columns of 11.5^o^  Saliency in first 5 fixations  Receiver Operating Characteristics (ROC)-analyses | USN patients without hemianopia had leftward saccades of lesser amplitude compared to non-USN patients with hemianopia (*g* = 1.7) and HC (*g* = 2.31). USN patients with hemianopia behaved the same compared to non-USN patients with hemianopia (*g* = 1.71) and HC (*g* = 2.08).  USN patients without hemianopia had rightward saccades of lesser amplitude compared to non-USN patients with hemianopia (*g* = 1.83) and HC (*g* = 2.18). USN patients with hemianopia behaved the same compared to non-USN patients with hemianopia (*g* = 1.36) and HC (*g* = 1.55).  No differences were found comparing N leftward saccades or percentage of leftward compared to rightward saccades.  All groups had prolonged fixations in the right hemifield compared to the left, no group differences.  USN without hemianopia produced fewer fixations in leftmost columns compared to non-USN with hemianopia (*g* = 3.52) and HC (*g* = 2.8) as did USN with hemianopia compared to non-USN with hemianopia (*g* = 1.87) and HC (*g* = 1.58). Both groups of USN had similar CFD.  In all groups first five fixations landed on more salient areas compared to random fixations.  Saliency and orientation maps were best to predict fixations in all groups. |
| Leyland et al. (2017) | Drawing faces in two conditions.  *Copy*: Two drawn faces are shown and must be copied on another piece of paper.  *Trace:* Two drawn faces are shown and subjects must draw upon the lines of shown faces. | Gaze (NA) | Peripersonal | Time spent in regions  N gazes  Accuracy of drawing %-wise (supplementary) | USN patients drew less accurate left halves of both the left (*g* = 1.83) and the right face (*g* = 0.94), compared to the right halves during copying.  USN patients drew less accurate the right half of the left face during Trace compared to Copying (*g* = 1.55).  USN patients were the only group showing differences in proportional time spent on halves of faces across Copy and Trace condition.  USN spent less time gazing on left halves of both the left (*g* = 4.47) and the right (*g* = 1.47) face in copying.  USN spent less time gazing on the left half of the left face compared to the left half of the right face (*g* = 1.65) and spent less time gazing on the right half of the left face compared to the right half of the right face (*g* = 1.56) in tracing. |
| Machner et al. (2012) | *Free-viewing* videos and photos (screenshots from video) from urban areas.  *Visual search* during videos looking for a specific target (static or dynamic) in video (left or right hemifield).  Stimuli analyzed for: brightness, color, static contrast and dynamic contrast. | Fixations (>60 ms)  Saccades (>.5^o^ position change in 2 consecutive samples between fixations) | Extrapersonal | N saccades  %-wise saccades in left hemifields and directed left.  Amp. of saccade in each hemifield and direction.  Fixations duration in each hemifield.  Median x-axis fixation position | USN had fewer saccades in free-viewing compared to non-USN (*g*= 2.47) and HC (*g* = 2.46) independently of condition. Further USN had fewer saccades in the left hemifield compared to non-USN (*g* = 3.55) and HC (*g*=4.44) independently of condition.  N left hemifield saccades changes across conditions for HC only, having fewer in search.  No group differences regarding leftward saccades.  USN had saccades of lesser amplitude in general compared to non-USN (*g* = 1.99) and HC (*g* = 1.95), and only USN had changes in amplitude with larger leftward saccades than rightward one (*g* = 0.54)  USN had longer fixations than non-USN (*g* = 1.52) and HC (*g* = 1.44), and their fixations in the right hemifield than those in the left hemifield (*g* = 1.27).  USN patients’ median fixation position on the x-axis was further right than in non-USN (*g* = 2.25) and HC (*g* = 3.02).  In free-viewing videos USN fixated on areas of low dynamic right of their median gaze position and areas of higher dynamic left of the same point.  In free-viewing photos USN fixated on areas of lower contrast than non-USN and HC but higher than random.  In search during videos USN fixated on areas of higher static contrast than non-USN, HC and random and on areas of higher dynamic contrast in the right hemifield compared to non-USN and HC. |
| Machner et al. (2018) | *Visual search* during computerizes task, searching a desk with 30 distractor items for a target paperclip in a specific color (red/blue) for max. 12 seconds. Target present in 80% of trials. Present in one of four columns.  (Posner task is not considered) | Gaze (NA) | Extrapersonal | Horizontal gaze distribution in cumulated time.  Center of fixation (CoF, median x-position of gaze)  Field of exploration (FoE, area of 50% of all gaze points around median point). | USN patients scoring severe on CBS^8^ had a CoF further right than moderate USN (*g* = 1.76) non-USN (*g* = 2.27) and HC (*g* = 4.66), No difference in comparing moderate USN and non-USN. Compared to HC, non-USN had a CoF further right (*g* = 2.8).  USN patients scoring severe on CBS had smaller FoE than non-USN (*g* = 1.82) and HC (*g* = 1.67) but not compared to moderate USN. Moderate USN did not differ compared to non-USN or HC. |
| Ohmatsu et al. (2019) | *Free-viewing* 6 naturalistic photos shown for 5 seconds each in a pseudorandomized order either a single photo in either original or flipped condition, or two identical photos mirrored around the vertical axis. | Fixations (>100 ms)  Gaze (NA) | Extrapersonal | N fixations  Total gaze distance  CFD %-wise in 16 bins of 1.85^o^  Gaze onset  Horizontal gaze velocity, right- and leftward  Gaze shift from initial to search. | USN patients’ gaze did always tend to search right side of an image regardless of it was left/right dominant of features, whereas HCs gaze followed the dominant feature. (Only plots available). Non-USN had different distribution from HC in 3/6 images (chi-square).  USN patients’ gaze shifted further right after the first 50 ms of search compared to non-USN (*g* = 7.27) and HC (*g*=8.74).  USN patients’ fixations position tended to shift more to the right compared to HC and non-USN. No difference for non-USN and HC (Only plots available).  USN and non-USN were slower than HC to initiate search (Only plots available).  USN had more fixations in the right hemifield and fewer in the right hemifield compared to non-USN and HC. (Only plots available)  A more rightward gaze shift in ^o^ was associated with shorter trajectory in cm for USN (*r*=-.53) and non-USN (*r*=*-.26*) but not HC. |
| Paladini et al. (2019) | *Free-viewing* 24 photos of natural scenes or urban public places for 7 seconds as either original or mirrored photo. Saliency maps calculated for each photo (intensity, colour and orientation) | Fixations (>100 ms) | Extrapersonal | N fixations  N fixations in hemifields  Counting CFD in 10 columns  Rate of re-fixations (inside Euclidean-distance ≤32 pixels)  Hemifield of first re-fixation  N fixation prior firs re-fixation  Rate of perseverative fixations (re-fixation of re-fixation) | USN patents had a rightward skewed distribution revealed by a correlation between horizontal position and number of fixations (*r*=*.55*), not found in HC.  Higher re-fixation rate in right than left hemifield for USN (*z* = *4.16*).  USN had more refixations in the right hemifield (*g* = 0.67) and fewer in the left hemifield (*g* = -0.99) in general compared to HC.  USN had more refixations in the right hemifield compared to the left hemifield (*g* = 1.028) in general. No difference for HC.  USN had more refixations in the right hemifield (*g* = 0.99) and fewer in the left hemifield (*g* = -0.58) in early search compared to HC.  USN had more refixations in their right hemifield compared to the left (*g* = 1.08) and HC had to opposite (*g* = -0.36) in early search.  USN had a mean fixation point further left (5.53 degrees) compared to HC (-0,12 degrees) (Only means available).  Lower perseveration rate for HC than USN (*z*=*-1.98*). (No further data available).  USN had higher probability for a first re-fixation to occurring in right hemifield. USN were more likely to have re-fixations than HC in early visual search (no means available).  USN produced fewer fixations prior to first re-fixations than HC (*z*=*-2.67*) (No further data available).  Re-fixations of USN occurred on more salient features than new fixations (*z*=*2.75*) in left hemifield only. For HC only in right hemifield (*z*=*3.13*). Both new (*z*=*-2.83)* and re-fixations (*z*=*-3.62*) occurred on more salient areas in left than right hemifield for USN no difference for HC (No further data available). |
| Primativo et al. (2015) | *Free-viewing* “Cookie-theft-image” from Boston Aphasia Battery with verbal description. No time limit.  *Single-word reading task* list of 42 7-letter words, letter-based analysis of left/middle/right omissions.  *Paragraph reading* of 5 texts with 5 lines analysing text- and word-based errors.  *Experimental saccade tasks* in overlap or gap condition, targets in either both or only right hemifield. | Fixations (NA)  Saccades (NA) | Extrapersonal | N fixations  Fixation duration  %-wise fixations in ROI^10^  Saccade amplitude  Saccade latency  Gaze time (ms) | Number and duration of fixations did not differ nor did saccade amplitude (Not possible to calculate g).  % fixations in ROI ranged from 75 to 89 in non-USN and for six USN from 80 to 87. Four USN ranged from 44 to 69 from beginning of search (Not possible to calculate g).  In single-word reading the four USN had more fixations and longer gaze time than remaining groups (Not possible to calculate g).  In paragraph-reading the four USN made more fixations than remaining groups, no differences on side (Not possible to calculate g).  In paragraph-reading the four USN had longer duration of fixations in the right side of the text (Not possible to calculate g).  Case-control experiments showed:  Differences in saccade latency and amplitude of saccade in both overlap and gap condition for two USN (Not possible to calculate g)  Differences were less constant in gap condition with only right hemifield targets for the two USN albeit still present (not possible to calculate g). |
| Ptak et al. (2009) | *Free-viewing* 20 colored photos in 1280x1024 pixels of natural scenes, architecture, man-made objects or regular patterns for 15 seconds. Photos roughly symmetrical without highly salient features.  Luminance, luminance contrast, chromatic contrast and edge information calculated for each photo. | Fixations (>100 ms)  Saccades (> 3^0^ amplitude; >50^0^/_sec_ velocity) | Extrapersonal | Fixation density in 32x26 grid of 1^0^ cells  Normalized fixation density  N saccades total  N saccades leftward/rightward  Saccade trajectory  Direction of first saccade | USN had saccades of lesser amplitude compared to non-USN (*g* = 1.31) and HC (*g*=1.75), non-USN and HC did not differ. As a result, USN had a shorter total saccade trajectory compared to non-USN (*g* = 1.29) and HC (*g* = 1.87).  USN mad fewer fixations in the left side (mean = 23.3%) compared to non-USN (mean = 60.5%) and HC (mean = 53.1%) (No further data available).  USN had a most frequent fixation point located further right (1 degree up, 6 degrees to the right) compared to non-USN and HC (both 1 degree up, and one degree right). USN had a midline located further right (5.6 degrees) than non-USN (-3.2 degrees) and HC (-2 degrees). (Only available in plots).  USN tended to fixate lesser on objects in photos compared to non-USN and HC (chi-square=12.2) (No further data available).  USN patients tended to direct the first saccade rightwards opposite non-USN and HC.  All groups were attracted by analyzed saliency features compared to random. (Only available in plot)  USN fixated on more luminant areas and areas with low edge content in left hemifield compared to HC and non-USN. No differences in right hemifield. (Only available in plots). |
| Serino et al. (2006) | Asked to read aloud 48 letter strings of either 9 or 11 letters, half the words are correctly spelled, half the words have had letters switched around. Tasked used as outcome measure for PA. Not all participants underwent ET. | Saccades (NA)  Gaze (NA) | Extrapersonal | Mean amplitude/landing position of first saccade (stimuli separated in columns of unknown width)  Gaze time (Left and right according to middle letter of string, unknown calculation of index). | After PA^11^ training USN produces a first saccade further to the left (-1.87 degree) than at baseline (-0.66 degree) whereas control USN did not differ from after cognitive training (0.02 degrees) and at baseline (0.26 degree). Patients who were better at adapting to prisms during week 1 produced a first saccade further to the left after PA (B_unstandardized_ = .78). Gaze time index varied in experimental group from baseline (.15) to post-PA (.30) but not in control group (.11 and .07) since the experimental group spent proportionally more time in the left hemifield after PA.  (Only mean values are reported). |
| Serino et al. (2007) | Asked to read aloud 48 letter strings of either 9 or 11 letters, half the words are correctly spelled, half the words have had letters switched around. Tasked used as outcome measure for PA. Not all participants underwent ET. | Saccades (NA)  Gaze (NA) | Extrapersonal | Mean amplitude/landing position of first saccade (stimuli separated in columns of unknown width)  Gaze time (Left and right according to middle letter of string, unknown calculation of index). | USN patients had a first saccade further left after PA (-2.05 degrees) than before (-5.8 degrees) and measured by a time index more time was spent in the left hemifield after PA (.28) than before (.16).  USN had better reading accuracy after PA (48%) than before (31%).  6 USN patients had follow-ups at 1 and 3 months showing persistent improvements in first saccade amplitude, time index and accuracy.  USN patients with pointing errors >1 degree (N=5) had lesser improvement on BIT^12^ than those who better adapted to PA (N=15).  USN with pointing errors >1 degree had a further right placement of the first saccade (-.23 degree) than the other patients (N=10, -1.69 degree).  (Only mean values are reported) |
| *Note:* ^1^Eye-tracking; ^2^Eye movements, ^3^Unilateral Spatial Neglect; ^4^cumulative fixation duration, ^5^Visual Field Deficit, ^6^Right Hemisphere Stroke Patients without USN, ^7^Healthy Control group, ^8^Catherine Bergego Scale, ^9^Line Bisection Task, ^10^Region of Interest, ^11^Prismatic Adaptation, ^12^Behaviroral Inattention Test  ^a^ = Values used for calculating effect sizes have been estimated from reading pixels, and SD has been calculated using reported SEM.  ^b^ = Assessed spaces are inferred from descriptions of stimuli and task instructions since it is not mentioned explicitly by any study. In some cases the adequate data was not available nor readable from provided tables or graphs. In such cases no Hedge’s g was calculated, and only effect sizes from main article were reported. | | | | | |
